# Spatial epigenomics reveals stromal niche-associated regulatory programs across pancreatic neuroendocrine tumor grades

**DOI:** 10.1016/j.isci.2026.117953

**Published:** 2026-10-19

**Authors:** Dejiang Wang, Xiangjun Di, Fu Gao, Gaocai Li, Liyun Lin, Shujie He, Di Zhang, Junyi Xu, Yu Jin, Yuanxin Liang, Michael Cecchini, Jill Lacy, John Kunstman, Pamela Kunz, Jing Du, Yang Liu

**Affiliations:** 1 Department of Pathology, Yale School of Medicine, New Haven, CT, USA; 2 Department of Neurology, Yale School of Medicine, New Haven, CT, USA; 3 Department of Biomedical Engineering, Yale University, New Haven, CT, USA; 4 Department of Internal Medicine (Medical Oncology), Yale School of Medicine, New Haven, CT, USA; 5 Department of Surgery, Yale School of Medicine, New Haven, CT, USA

**Keywords:** NF-PanNETs, spatial ATAC-seq, single-nucleus ATAC-seq, chromatin accessibility, tumor microenvironment, cancer-associated fibroblasts, tumor progression

## Abstract

Non-functional pancreatic neuroendocrine tumors (NF-PanNETs) account for the majority of pancreatic neuroendocrine neoplasms and exhibit substantial clinical and biological heterogeneity, yet their epigenetic regulation and spatial architecture remain poorly understood. Here, we present an integrative analysis of NF-PanNETs across multiple tumor grades using single-nucleus advent of transposase-accessible chromatin using sequencing (snATAC-seq) and spatial ATAC-seq. snATAC-seq identified grade-associated chromatin accessibility programs characterized by distinct transcription factor activities, including HNF1A and FOXA2 in low-grade tumors, ETS family factors in intermediate-grade tumors, SNAI-associated regulatory programs in high-grade tumors, and CEBPB- and TOX-associated immune states. Spatial ATAC-seq revealed spatially coordinated tumor-stroma interactions, with stroma-proximal regions enriched for MYC/FOX-associated proliferative programs and distal regions exhibiting SNAI-enriched invasive programs. Together, our study provides a spatially resolved epigenomic atlas of NF-PanNETs across tumor grades, offering a resource for understanding epigenetic heterogeneity and generating hypotheses regarding regulatory programs and tumor-stroma interactions for future functional and clinical investigation.

## Highlights

• snATAC-seq defines grade-associated regulatory programs in NF-PanNETs
• Immune and fibroblast states show distinct chromatin regulatory programs
• Spatial ATAC-seq maps grade-associated tumor states in native tissue
• Tumor accessibility states associate with myCAF-rich spatial regions

## Graphical abstract

**Figure:**
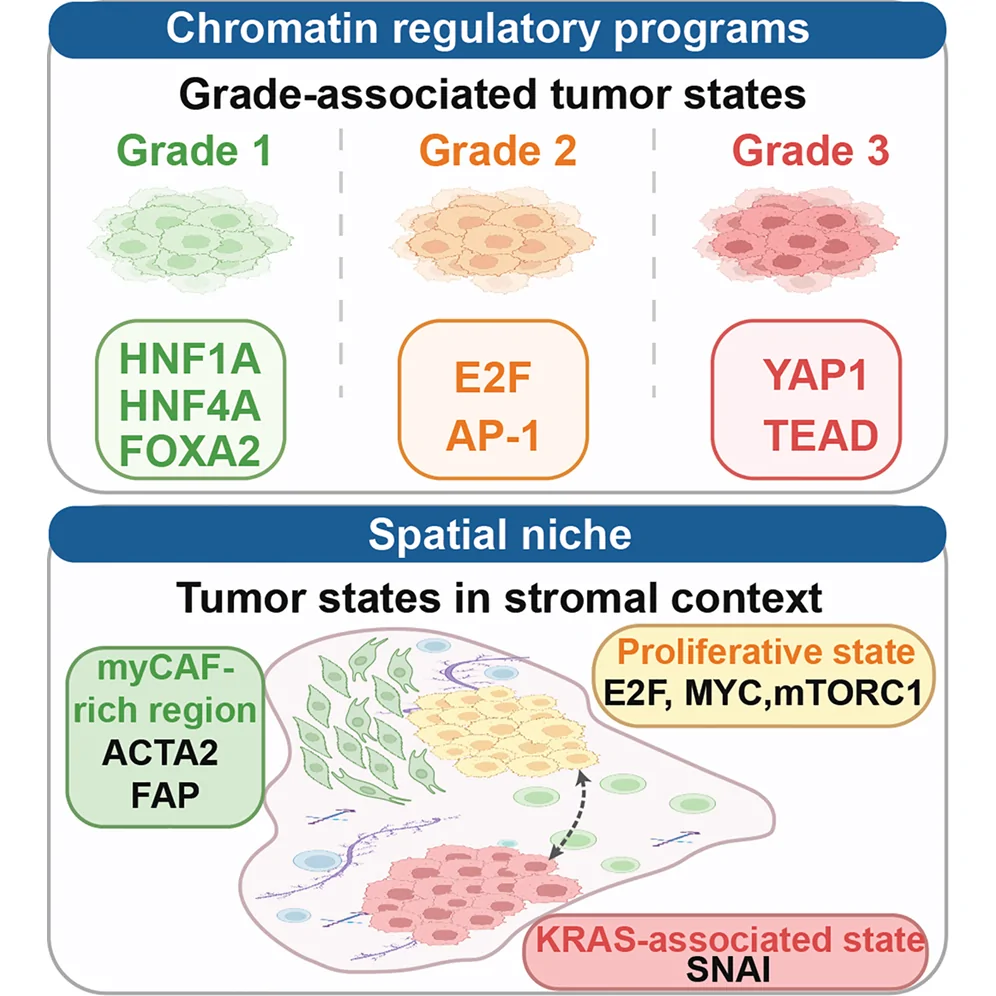

## Teaser

Cancer; Genomics; Systems biology

## Introduction

Pancreatic neuroendocrine tumors (PanNETs) represent the second most common type of epithelial neoplasm in the pancreas. PanNETs are defined as heterogeneous malignancies originating from the neuroendocrine precursor cells or neuroendocrine transdifferentiation of epithelial cell from pancreas.^1^ They are classified into functional and non-functional (NF-PanNETs) subtypes according to the presence of clinically manifest hormonal hypersecretion syndromes.^2^ NF-PanNETs account for 60%–90% of PanNETs and are increasing in incidence and prevalence over time owing to advances in molecular imaging technologies.^3^ Although some NF-PanNETs may present with a relatively indolent course, it is critical to recognize their intrinsic malignancy and variable capacity for invasion. Approximately 40%–80% of patients with NF-PanNETs have distant metastasis at the time of diagnosis, most frequently involving the liver (40%–93%), and exhibit significantly worse prognosis.^4^^,^^5^ Currently, clinical treatment decisions are primarily guided by tumor grade and stage. Systemic therapies,^6^ such as chemotherapy^7^ (e.g., temozolomide), targeted therapy^8^ (e.g., mammalian target of rapamycin [mTOR] inhibitor), hormone therapy^9^^,^^10^ (e.g., somatostatin analogs), and peptide receptor radionuclide therapy,^11^^,^^12^ have showed clinical benefits on the progression-free survival; however, there is limited impact on the overall survival. Therefore, a comprehensive understanding of the molecular mechanisms driving the progression of NF-PanNETs is imperative for advancing clinical management and therapeutic strategies.

Genomic mutation landscape and dysregulated signaling pathways in NF-PanNETs have been characterized by whole-genome/exome sequencing and whole transcriptomic sequencing.^13^^,^^14^^,^^15^^,^^16^ NF-PanNETs frequently harbor mutations in the DAXX/ATRX, MEN1, and mTOR pathway genes.^17^ However, sporadic cases also exhibit alterations in genes such as TP53, CDKN2A, RB1, and KRAS, highlighting the molecular complexity and heterogeneity of NF-PanNETs.^18^^,^^19^ While genetic mutations play a critical role in the diagnosis and treatment of NF-PanNETs, growing evidence underscores the contribution of epigenetic modifications in shaping their complex signaling landscape.^20^ Epigenomic studies can provide critical insights into regulatory state transitions underlying both normal cellular functions and pathological reprogramming.^21^ In particular, the advent of transposase-accessible chromatin using sequencing (ATAC-seq) has enabled the genome-wide profiling of chromatin accessibility, offering a powerful approach to map *cis*- and *trans*-regulatory activity during tumor progression.^22^^,^^23^ However, bulk ATAC-seq captured an averaged chromatin accessibility profile, obscuring the genetic and regulatory heterogeneity among tumor subpopulations within the sample.^23^^,^^24^ Single-cell/nucleus ATAC-seq (sc/snATAC-seq) offers novel insights into regulatory state characterization at single-cell resolution but loses spatial context during sample processing.^25^ Notably, recent advances in spatial ATAC-seq now allow chromatin accessibility profiling within intact tissue architecture, restoring spatial context while retaining high-resolution epigenomic data.^26^^,^^27^ This enables the mapping of epigenetic states to specific anatomical locations and microenvironmental niches, thereby improving our understanding of how spatial interactions influence cell identity, plasticity, fate decisions, and tumor-microenvironment dynamics. Therefore, extending the study from isolated single cells to multicellular hubs of interacting cells allows for more refined analyses of phenotypic heterogeneity and regulatory programs, beyond what sc/snATAC alone can reveal.

In this study, we performed an integrative chromatin accessibility analysis using snATAC-seq and spatial ATAC-seq to map the epigenetic landscape of treatment-naïve NF-PanNETs across tumor grades. Our findings identify grade-associated transcriptional regulatory programs and spatially organized tumor microenvironmental niches associated with cellular heterogeneity, providing a foundation for future studies investigating molecular stratification and potential therapeutic vulnerabilities.

## Results

### Chromatin accessibility and cell lineage determination of NF-PanNET patient samples

We performed snATAC-seq using the Chromium platform (10x Genomics) on primary tumor specimens from nine untreated patients with NF-PanNETs, generating a total of 38,198 nuclei. In addition, spatial ATAC-seq was performed on twelve NF-PanNET specimens, six of which were matched to those analyzed by snATAC-seq (Figure 1A). The snATAC-seq data were filtered using thresholds of ≥ 500 unique nuclear fragments per nucleus and a transcription start site (TSS) enrichment score ≥ 3 to exclude low-quality nuclei (Figure S1A). Across all snATAC-seq samples, we identified 62,896 accessible chromatin regions (ACRs), the majority of which were located in promoter (46.3%), intronic (29.69%), and distal intergenic (18.98%) regions.

**Figure 1:**
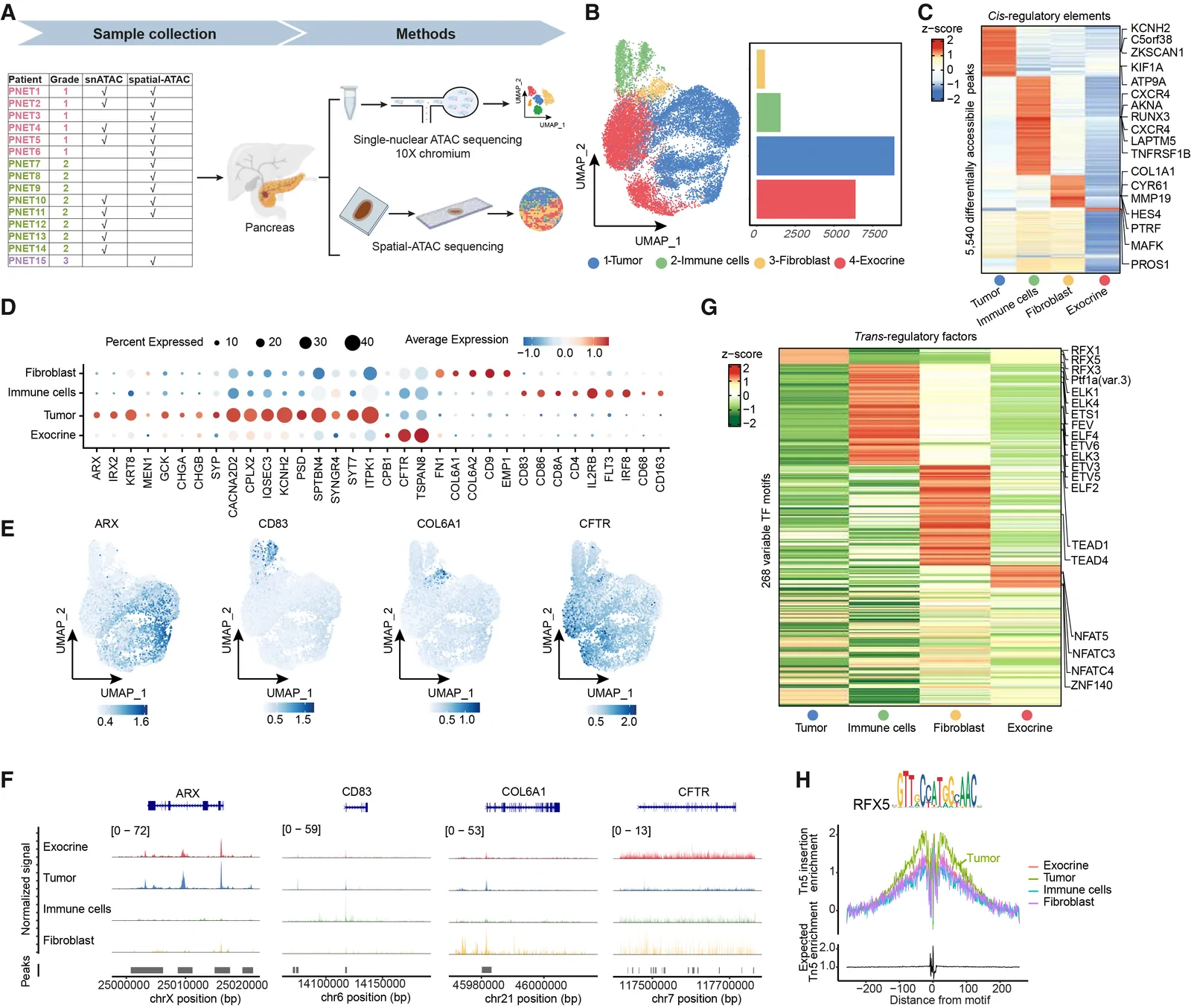
Chromatin accessibility analysis of cells across NF-PanNETs patients (A) Overview of the NF-PanNET cohort showing tumor grade and the availability of snATAC-seq and spatial ATAC-seq data for each sample. (B) UMAP projection of snATAC-seq profiles showing the major annotated cell populations. Right, bar plot showing the number of nuclei in each population. (C) Heatmap of cell-type-specific DACRs. Color indicates the z-scaled average normalized accessibility across cell populations. (D) Dot plot showing gene activity scores of representative lineage marker genes. Dot size represents the percentage of nuclei with detectable gene activity, and color indicates the average gene activity score. (E) UMAP projections of gene activity scores for selected lineage marker genes. (F) Genome browser coverage plots showing chromatin accessibility at representative marker gene loci across cell populations. (G) Heatmap showing differential TF motif activity across cell populations based on chromVAR deviation scores. Color indicates the z-scaled average chromVAR deviation score. (H) TF footprinting analysis of RFX5 across cell populations.

Unsupervised clustering identified four major cell populations—tumor, immune, fibroblast, and exocrine cells—based on their chromatin accessibility profiles (Figure 1B; Figure S1B). Across these populations, we identified 5,540 cell-type-specific differentially ACRs (DACRs). Annotation based on genes neighboring cluster-specific *cis*-regulatory elements revealed distinct cellular components of the tumor microenvironment (TME) (Figure 1C). For example, cluster 1 showed increased accessibility near tumor-associated genes, including KCNH2 and KIF1A. KCNH2 has been reported to be upregulated in pancreatic cancer and associated with cell proliferation, migration, invasion, and epithelial-mesenchymal transition (EMT),^28^ whereas KIF1A has been implicated in neuroendocrine differentiation.^29^ Cluster 2 was enriched for ACRs near immune-related genes, including CXCR4, RUNX3, and LAPTM5. Cluster 3 showed increased accessibility near fibroblast-associated genes, including COL1A1 and MMP19, supporting its fibroblast identity. We next generated a gene activity score matrix and further annotated cell populations based on the activity of canonical lineage markers (Figure 1D). Tumor cells showed increased gene activity for CHGA, CHGB, CACNA2D2, and SYT7, whereas fibroblasts were characterized by FN1, COL6A1, and COL6A2. Immune cells showed increased activity of markers including CD86, CD4, and IL2RB, whereas exocrine cells were characterized by CFTR and TSPAN8.^30^ Projection of representative marker gene activity scores onto the snATAC-seq uniform manifold approximation and projection (UMAP) further supported the cell identities inferred from *cis*-regulatory element accessibility (Figures 1E and 1F).

We next assessed TF-associated (Transcription Factor-associated) regulatory activity using chromVAR deviation scores calculated from accessible regions containing transcription factor binding motifs.^31^ Tumor cells showed increased deviation scores for RFX-family motifs, whose members have previously been implicated in tumor-associated regulatory processes^32^ (Figure 1G; Figure S1E). Consistent with this observation, TF footprinting analysis showed distinct accessibility profiles surrounding RFX binding motifs in tumor cells (Figure 1H). Immune populations showed increased deviation scores for ETS-family motifs, consistent with the established roles of ETS-family transcription factors in multiple immune lineages, including T cells, B cells, macrophages, neutrophils, and dendritic cells^33^ (Figure 1G). Finally, analysis of peak genomic annotations and 15-state ChromHMM annotations showed that accessible regions in immune cells and fibroblasts were frequently associated with promoter and enhancer states, consistent with active *cis*-regulatory landscapes within these tumor microenvironmental populations (Figures S1C and S1D).

### Characterization of chromatin accessibility alterations across tumor grades

To investigate chromatin accessibility dynamics across tumor grades in NF-PanNETs, we isolated tumor cells from snATAC-seq data. Using latent semantic indexing (LSI) and UMAP for dimensionality reduction and visualization, we identified nine tumor subclusters (Tumor_1 to Tumor_9; Figure 2A; Figure S2A), each defined by distinct chromatin accessibility profiles and gene activity signatures (Figures 2B and 2C). Notably, the distribution of these subclusters differed markedly between tumor grades. Grade 1 tumors were predominantly represented by Tumor_1 to Tumor_4, whereas grade 2 tumors were enriched for Tumor_5 to Tumor_9. Previous work in poorly differentiated neuroendocrine carcinomas identified five transcription factor-defined molecular subtypes characterized by ASCL1, NEUROD1, HNF4A, POU2F3, and YAP1 expression.^34^ We therefore examined whether related regulatory programs could be detected across the nine tumor clusters in our well-differentiated NF-PanNET cohort using gene activity of the core transcription factors, subtype-associated module scores, and chromVAR motif activity (Figure S3). Tumor_1–Tumor_3, which were predominantly associated with grade 1 tumors, showed overlapping NEUROD1- and HNF4A-associated features, with Tumor_2 additionally displaying an ASCL1-associated component. Tumor_5, enriched in grade 2 tumors, showed the clearest POU2F3-associated profile. Other clusters exhibited weaker or mixed subtype-related patterns, indicating that the NEC-derived classification does not map directly onto the NF-PanNET states identified here. Several grade 2 subclusters lacked canonical subtype markers but instead showed increased activity of genes such as PCSK1, PTEN, TRIM24, and TRIM28, indicating additional regulatory diversity beyond classical subtype definitions. Next, we performed pathway enrichment analysis, which revealed differential enrichment of biological programs across tumor subpopulations, including cell proliferation, EMT, and immune-related pathways (Figure S2B). Tumor_4, a grade 1 cluster, was enriched for proliferation-related gene sets, including E2F targets, G2M checkpoint, and MYC targets. Conversely, Tumor_1 and Tumor_5, which showed less prominent enrichment of proliferation-associated programs, were enriched for pathways such as KRAS signaling up and inflammatory response, both of which have been implicated in pancreatitis and pancreatic intraepithelial neoplasia (PanIN).^35^ DACRs across tumor subclusters and grades further underscored the epigenetic heterogeneity of NF-PanNETs (Figure 2D; Figure S2C). Genomic annotation and ChromHMM state analysis of DACRs revealed grade-associated differences in their genomic distribution, particularly in promoter and enhancer regions (Figures S2D and S2E).

**Figure 2:**
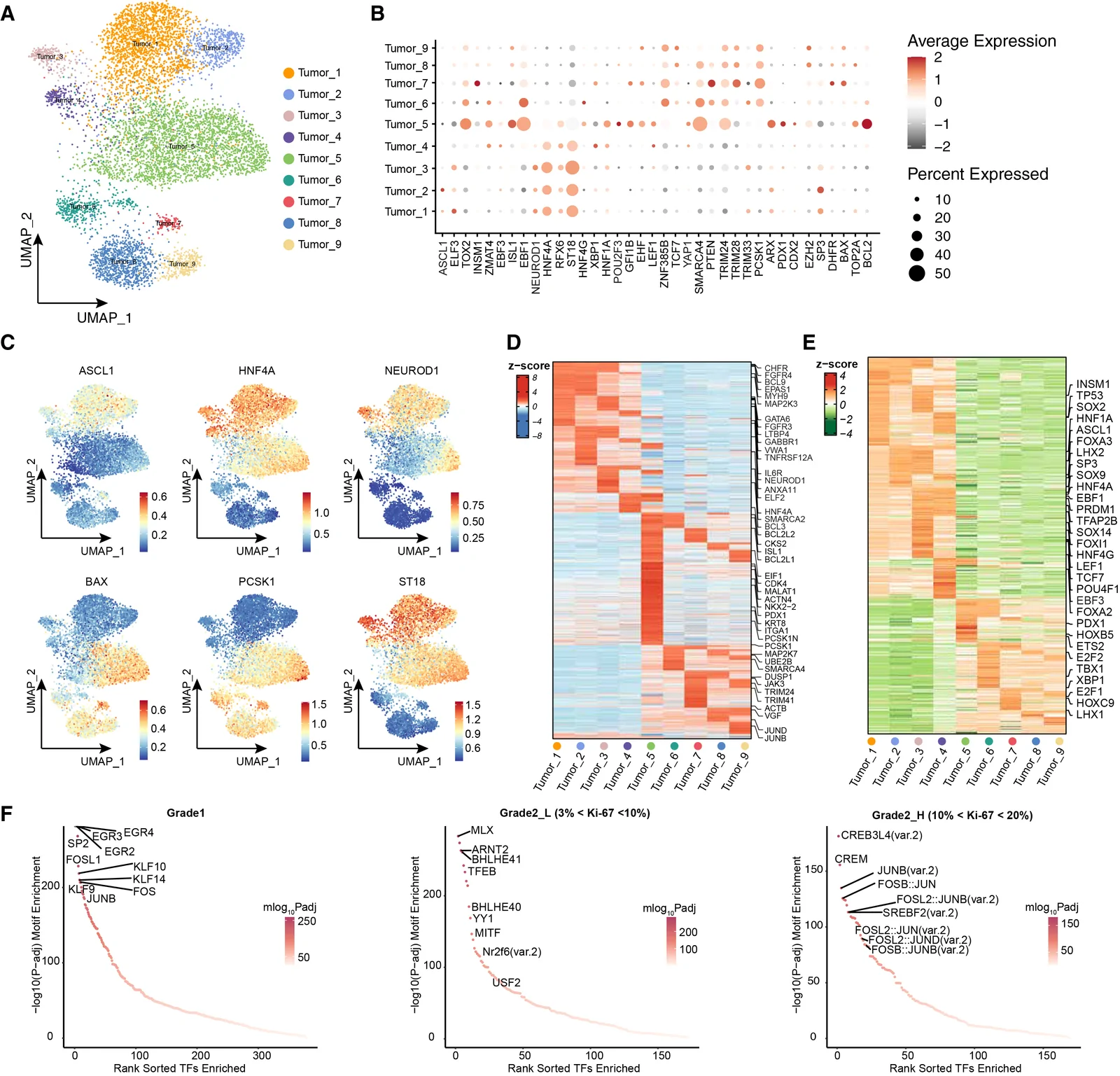
Tumor heterogeneity characterized by chromatin accessibility dynamics across tumor grades (A) UMAP projection of annotated tumor nuclei based on snATAC-seq profiles. (B) Dot plot illustrating gene activity scores of representative marker genes across tumor subclusters. Dot size indicates the percentage of nuclei with detectable gene activity; color reflects the average activity score. Dark red indicates higher gene activity scores, whereas dark gray indicates lower gene activity scores. (C) UMAP projections of gene activity scores for selected subcluster-associated marker genes. (D) Top DACRs identified for each tumor subcluster. Color indicates the z-scaled average normalized accessibility across tumor subclusters. (E) Differential chromVAR deviation scores across tumor subclusters. Color represents the z-scaled average chromVAR deviation score. (F) TF motif enrichment in grade-associated DACRs.

We next examined TF activity across tumor subclusters using chromVAR-derived deviation scores (Figure 2E). HNF1A, HNF4A, and HNF4G showed higher chromVAR motif deviation scores in grade 1 tumors, consistent with regulatory programs associated with differentiated neuroendocrine states. In contrast, grade 2 tumors showed higher chromVAR deviation scores for ETS2, E2F2, and E2F1 motifs, consistent with enhanced proliferative and cell-cycle-associated regulatory programs. Footprinting analysis further showed greater chromatin accessibility surrounding HNF1A binding motifs in grade 1 tumors compared with grade 2 tumors (Figure S2F). To further characterize the regulatory features associated with these accessibility differences, we performed motif enrichment analysis on DACRs (Figure 2F; Figure S2G). Motifs for KLF and EGR family members were enriched in grade 1 tumors, while TFEB and YY1 motifs predominated in low-proliferative grade 2 tumors (Grade2_L, Ki-67 3%–10%). In contrast, AP-1 motifs were significantly enriched in highly proliferative grade 2 tumors (Grade2_H, Ki-67 10%–20%). Given the established involvement of AP-1 family factors in proliferation, differentiation, apoptosis, angiogenesis, and invasion,^36^ AP-1 motif enrichment is consistent with a distinct proliferative and stress-responsive regulatory landscape in Grade2_H tumors.

### Transcription factor regulatory states along inferred tumor chromatin trajectories

Having identified tumor-subcluster-associated TFs that differed across histological grades, we next asked whether these regulatory states could be organized along a continuous chromatin accessibility trajectory. We therefore performed trajectory inference across all tumor cells. The resulting pseudotime ordering revealed a continuum in which grade 1 tumor cells were enriched toward one end and grade 2 tumor cells toward the opposite end (Figure 3A). The pseudotime trajectory thus captures a graded continuum of chromatin regulatory states associated with histological grade.

**Figure 3:**
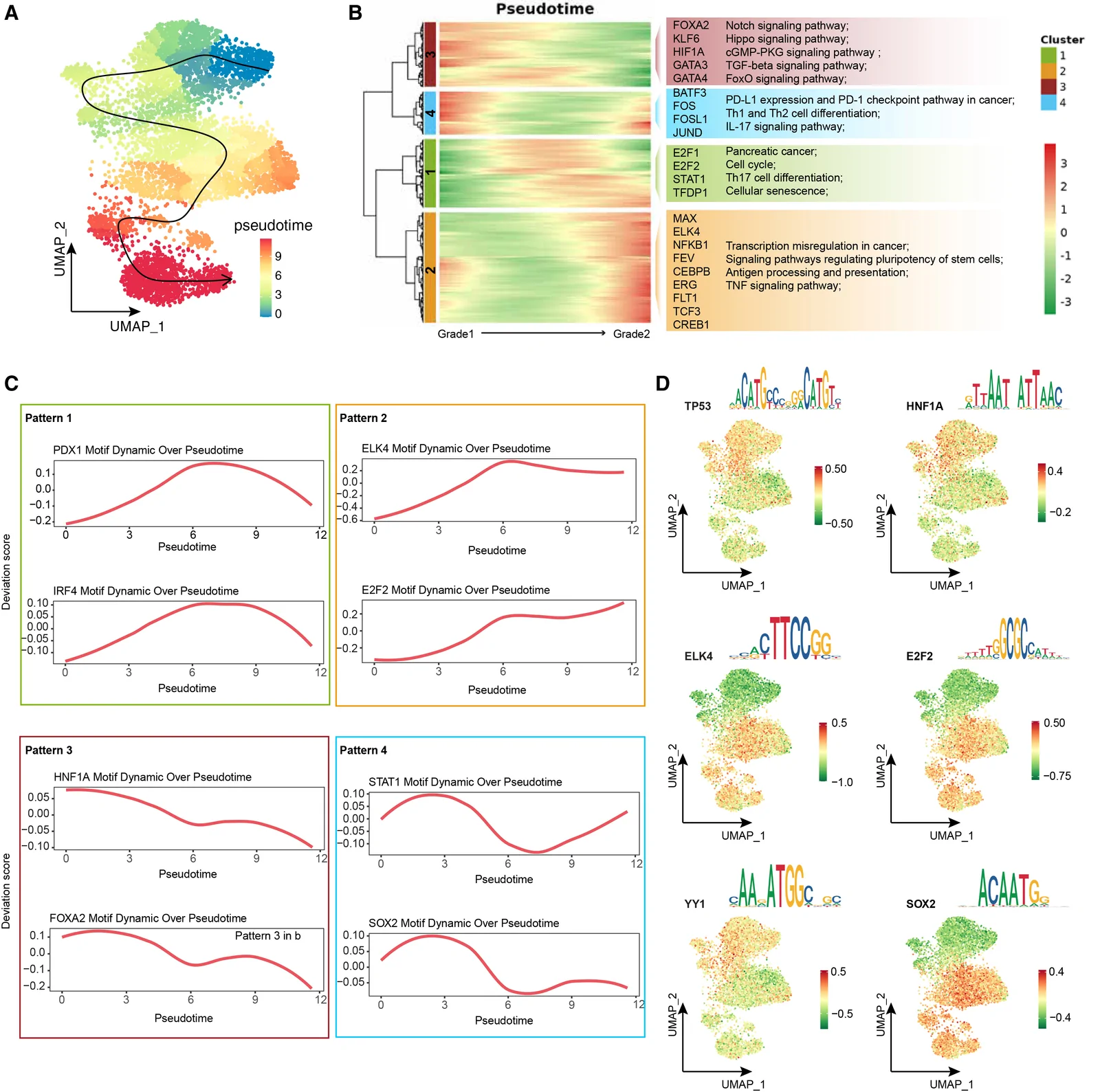
Grade-associated transcription factor regulatory states along an inferred chromatin accessibility trajectory (A) Pseudotime trajectory of tumor chromatin states inferred from snATAC-seq profiles. (B) Heatmap showing changes in chromVAR-derived TF deviation scores along the inferred pseudotime trajectory. The right image highlights representative TFs and enriched pathways associated with each activity pattern. (C) Line plots showing representative TF deviation-score patterns along pseudotime. (D) UMAP projections of chromVAR deviation scores for selected TFs across tumor cells.

We next examined changes in TF motif-associated activity along the inferred trajectory using chromVAR-derived TF deviation scores. Unsupervised clustering of the smoothed scores identified four major activity patterns (Figures 3B and 3C). Patterns 1–2 included TFs whose chromVAR deviation scores increased along pseudotime, such as ELK4 and E2F2, with associated target genes enriched in cell-cycle-related pathways, MAPK/ETS signaling, TNF signaling, and transcriptional regulation in cancer. In contrast, pattern 3 contained TFs whose chromVAR deviation scores decreased along pseudotime, including FOXA2 and HNF1A, with associated target genes enriched in Notch, Hippo, cGMP-PKG, TGF-β, and FoxO pathways. Pattern 4 showed a non-monotonic profile, with reduced chromVAR deviation scores in intermediate pseudotime states. The associated target genes were enriched for immune-related pathways, including PD-L1/PD-1 signaling, Th1/Th2 differentiation, and IL-17 signaling. Representative TFs with distinct activity patterns were further visualized along pseudotime and projected onto the snATAC-seq UMAP (Figures 3C and 3D). HNF1A and FOXA2 showed higher chromVAR deviation scores toward the grade 1-associated end of the trajectory, whereas ELK4, E2F2, and SOX2 showed higher deviation scores toward the grade 2-associated end. Together, these findings identify distinct grade-associated TF regulatory programs among NF-PanNET tumor states without implying a direct temporal or causal transition between grades.

### Chromatin landscape of intratumoral immunity

To characterize the immune microenvironment in NF-PanNETs, we subdivided immune cells into myeloid and lymphoid compartments (Figures 4A and 4B; Figure S4A). We classified myeloid cells by their high gene activity scores for ITGAM, CD68, and CD163. Within the lymphocyte compartment, distinct subsets were identified, including T cells (CD8A and CD8B), B cells (CD19 and CD79A), and natural killer (NK) cells (SPON2, IL2RB, and KLRD1). NK cells, as key players in cancer immunosurveillance, exert anti-tumor functions through natural cytotoxicity and antibody-dependent cellular cytotoxicity.^37^ Kyoto Encyclopedia of Genes and Genomes (KEGG) pathway enrichment analysis of NK cell-associated DACRs revealed enrichment of pathways including NK cell-mediated cytotoxicity, Fc gamma R-mediated phagocytosis, antigen processing and presentation, and apoptosis (Figure 4C). Furthermore, NK cells displayed increased chromatin accessibility at the promoter region of the IL2RB locus (Figure S4B). In contrast, B cells contribute to adaptive immunity primarily through antigen presentation and antibody secretion.^38^ KEGG pathway analysis of B cell-associated DACRs indicated enrichment of pathways related to the B cell receptor signaling pathway, NF-κB signaling, and cellular senescence.

**Figure 4:**
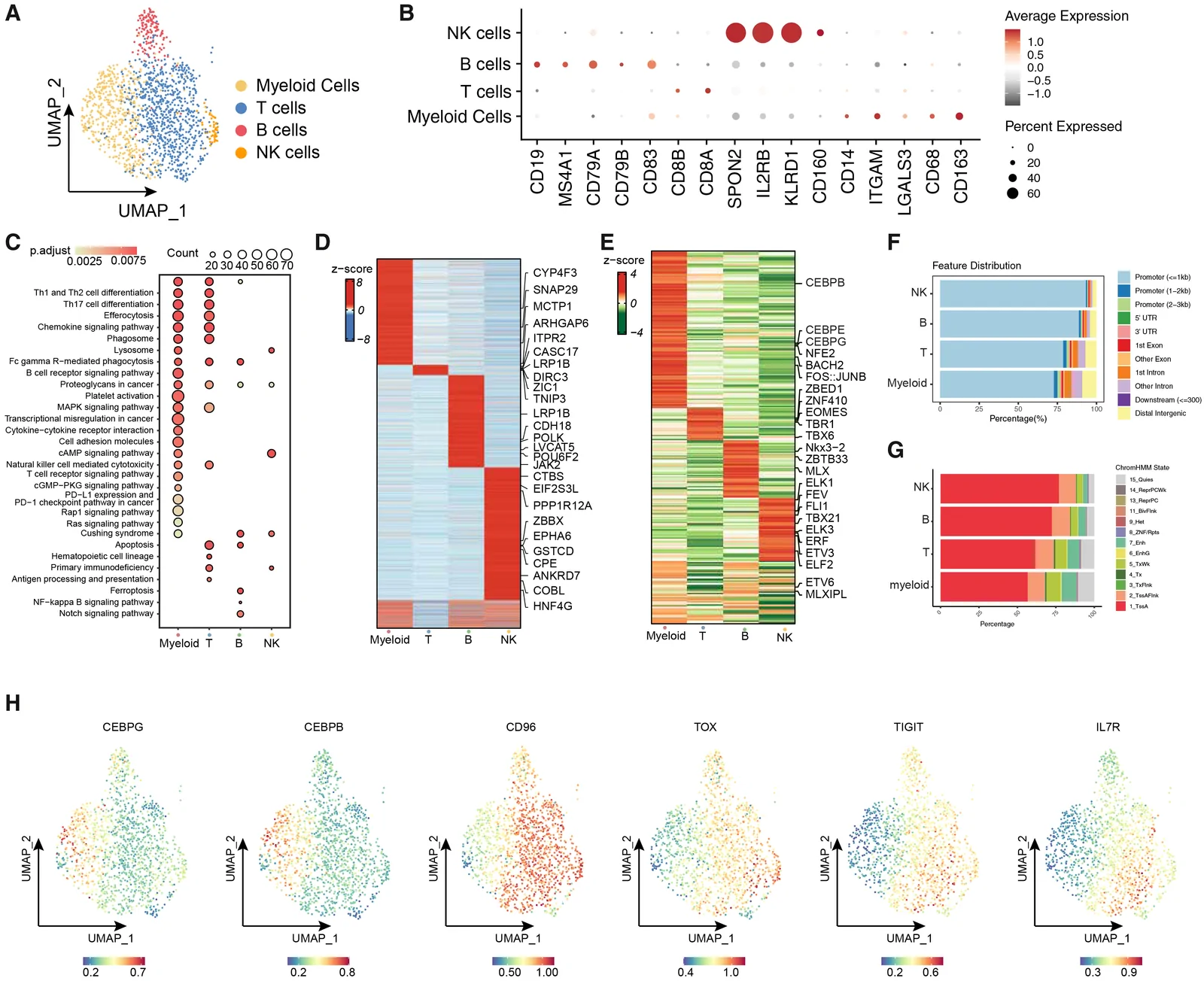
Chromatin landscape of intratumoral immunity in NF-PanNETs (A) UMAP projection of snATAC-seq profiles showing annotated immune cell subtypes. (B) Dot plot of gene activity scores for canonical marker genes across immune cell subtypes. Dot size represents the percentage of nuclei with detectable gene activity; color indicates the average gene activity score. Dark red indicates higher gene activity scores, whereas dark gray indicates lower gene activity scores. (C) Immunologic signature and KEGG pathway enrichment analysis of DACRs in each immune cell subtype. (D) Heatmap showing the top DACRs for each immune cell subtype. Color reflects the Z scored average normalized accessibility across immune cell subtypes. (E) Heatmap of chromVAR-derived TF deviation scores across immune cell subtypes. Color indicates z-scaled average chromVAR deviation scores. (F) Genomic feature distribution of immune subtype-specific DACRs, including promoter, enhancer, and intergenic regions. (G) ChromHMM state annotation of DACRs for each immune cell subtype. State definitions: TssA (active TSS), TssAFlnk (flanking active TSS), TxFlnk (transcription at gene 5′/3′), Tx (strong transcription), TxWk (weak transcription), EnhG (genic enhancers), Enh (enhancers), ZNF/Rpts (ZNF genes and repeats), Het (heterochromatin), TssBiv (bivalent/poised TSS), BivFlnk (flanking bivalent TSS/enhancer), EnhBiv (bivalent enhancer), ReprPC (repressed PolyComb), ReprPCWk (weak repressed PolyComb), and Quies (quiescent/low). (H) UMAP projections of gene activity scores for representative marker genes across immune subtypes.

Compared with NK and B cells, myeloid cells showed broader enrichment across multiple immune- and signaling-related pathways.^39^ Pathway analysis revealed that myeloid cells were enriched in chemokine signaling, MAPK signaling, cytokine-cytokine receptor interaction, cAMP signaling, cGMP-PKG signaling, Rap1 signaling, and Ras signaling pathways (Figure 4C), highlighting the diverse regulatory programs associated with myeloid cells in the TME. Consistent with these pathway-level differences, analysis of cell-type-specific DACRs identified increased accessibility at several myeloid-associated loci (Figure 4D). Notable examples included CYP4F3, an enzyme involved in leukotriene B4 degradation and inflammation resolution,^40^ and SNAP29, a gene implicated in vesicle fusion and immune regulation.^41^ Myeloid-derived suppressor cells (MDSCs), which arise in contexts of chronic inflammation and advanced cancers, are known to drive immune evasion and tumor progression.^42^ MDSC-associated differentiation has been described within a two-signal framework: the first involves STAT3 activation and regulation by transcription factors including CEBPA, CEBPB, and IRF8^43^^,^^44^; the second engages the NF-κB pathway, stimulated by inflammatory mediators like TNF-α, various interleukins, and HMGB1.^45^^,^^46^ In our data, we observed increased C/EBP-family motif activity together with elevated gene activity scores for CEBPB, CEBPE, and CEBPG in myeloid cells, suggesting the presence of MDSC-like regulatory features within the myeloid compartment (Figures 4E and 4H). Genomic annotation and ChromHMM segmentation of ACRs further revealed that myeloid- and T cell-associated DACRs were less frequently annotated to promoter and enhancer states compared with those of other immune subtypes (Figures 4F and 4G), indicating distinct regulatory architectures among immune cell populations within the TME.

To further dissect myeloid heterogeneity, we reclustered myeloid cells into four subsets (Figure 5A; Figure S4C). We then assessed the gene activity of markers associated with M1, M2a, M2c, and tumor-associated macrophages (TAMs) (Figure S4D). Interestingly, none of the four macrophage subsets conformed strictly to canonical M1/M2 polarization, suggesting more complex or intermediate activation states. This complexity is also consistent with previous single-cell studies of PanNETs, which identified heterogeneous immune populations and dynamic myeloid programs within primary and metastatic lesions.^13^^,^^47^ In our data, macrophage subset 1 showed increased gene activity at IRF8 and ZBTB46 (Figure 5B), suggestive of an early myeloid differentiation or antigen-presenting program. IRF8 has established roles in mononuclear phagocyte differentiation and TAM antigen presentation, although its functional consequences can vary according to tumor context. Macrophage subset 3 showed higher gene activity for HMGB1 and CCL18 (Figure 5B), together with inflammatory and suppressive pathway enrichment, consistent with a more activated, tumor-associated state (Figure 5C). The subcluster-specific TF programs further support this interpretation. Higher SREBF1/2-associated motif activity in macrophage 0 suggests lipid-metabolic reprogramming, a recognized feature of TAM adaptation, whereas the HOX- and TEAD-family motif activities observed in macrophage 3 indicate additional regulatory heterogeneity not encompassed by classical polarization categories (Figure 5D). Collectively, these findings extend previous transcriptomic observations by providing a chromatin-regulatory view of macrophage diversity in NF-PanNETs and support the presence of multiple intermediate or specialized myeloid states potentially associated with immune regulation within the TME.

**Figure 5:**
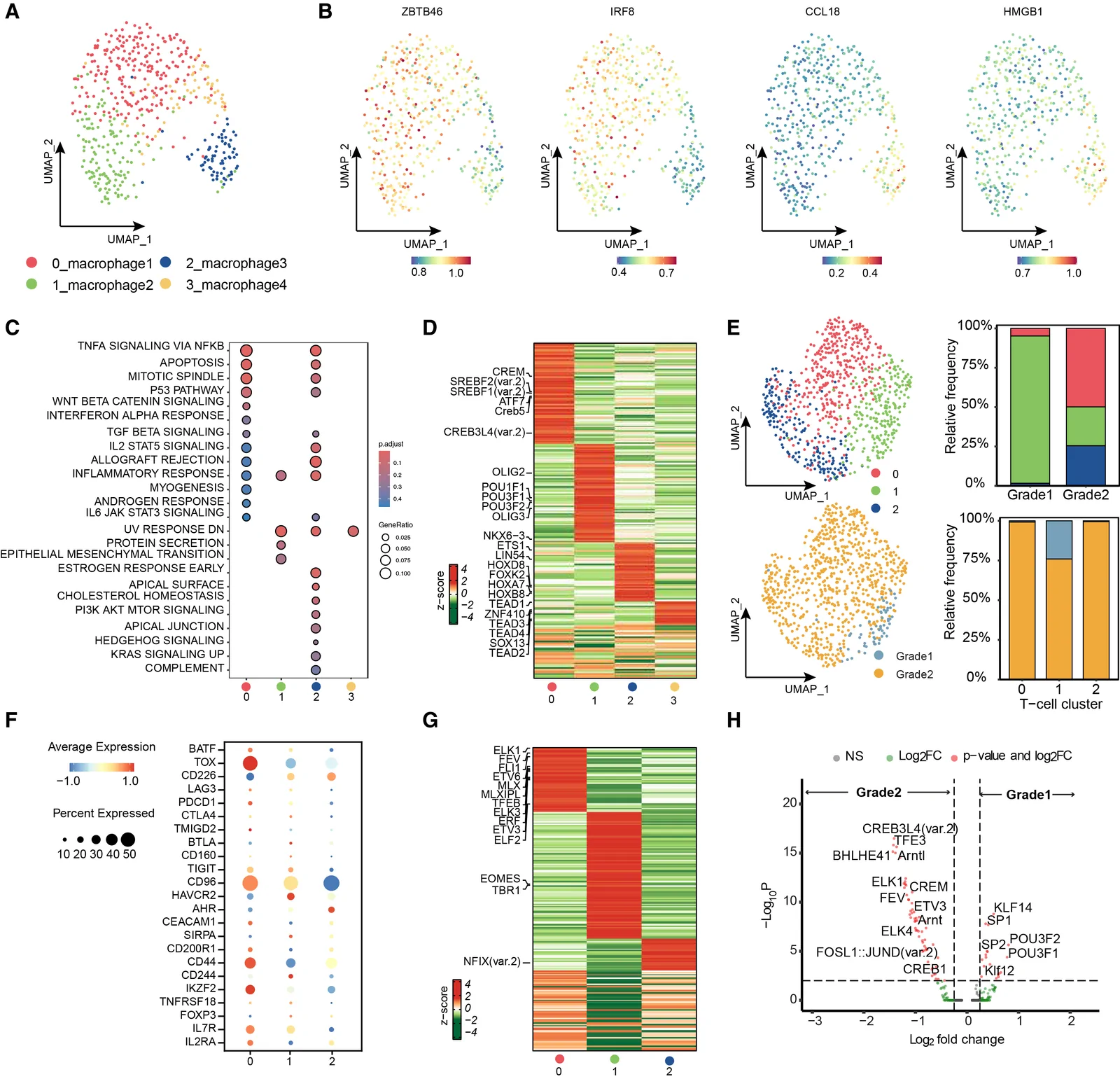
Epigenetic heterogeneity of macrophages and T cells in NF-PanNETs (A) UMAP projection showing annotated snATAC-seq profiles of macrophage subclusters. (B) UMAP projections of gene activity scores for representative marker genes across macrophage subclusters. (C) Enriched hallmark pathways associated with DACRs showing increased accessibility in each macrophage subcluster. Dot size reflects the gene ratio, and color indicates the adjusted *p* value. (D) ChromVAR-derived TF deviation scores across macrophage subclusters. Color represents the z-scaled average chromVAR deviation score. (E) UMAP projection showing the three T cell subclusters (left) and stacked bar plots showing their relative abundance in grade 1 and grade 2 tumors (right). (F) Dot plot of gene activity scores for marker genes of T cell subclusters. Dot size indicates the percentage of nuclei with detectable gene activity; color represents average activity score. Dark red indicates higher gene activity scores, whereas dark blue indicates lower gene activity scores. (G) ChromVAR-derived TF deviation scores across T cell subclusters. Color represents the z-scaled average chromVAR deviation score. (H) Volcano plot showing differential TF motif enrichment between grade 1 and grade 2 T cells.

To delineate the heterogeneity of T cells in the TME of NF-PanNETs, we selected cells showing gene activity for canonical T cell markers CD8A and CD8B, identifying three chromatin-defined T cell clusters (Figures 4B and 5E). Clusters 0 and 2 were predominantly enriched in grade 2 tumors, whereas cluster 1 was the major subset found in grade 1 tumors (Figure 5E), indicating a grade-associated difference in T cell cluster distribution. To further characterize these subsets, we examined gene activity scores of T cell subcluster markers and immune regulatory genes (Figure 5F). Notably, cluster 0 displayed elevated gene activity scores for TOX, CD96, CD44, and IKZF2, consistent with an exhaustion-associated regulatory state. Pathway enrichment analysis of cluster 0 further supported this interpretation, with significant enrichment of the Hedgehog signaling pathway (Figure S4E), a pathway previously implicated in immune suppression and immunosuppressive tumor microenvironments.^48^ However, the exhaustion-associated features of cluster 0 did not fully align with classical exhaustion signatures. Specifically, key inhibitory receptors such as PDCD1 (PD-1) and CTLA4 did not show prominent gene activity. This observation is consistent with prior findings that PD-1/PD-L1 expression is generally low or absent in small intestinal and PanNETs.^49^ These findings suggest the presence of non-canonical exhaustion- or immune suppression-associated regulatory features in the NF-PanNET microenvironment. Moreover, we observed higher gene activity scores for several non-classical immune checkpoint-associated genes in grade 2 tumors (Figure S4F).

To characterize T cell regulatory heterogeneity, we compared chromVAR-derived TF deviation scores across the three T cell clusters (Figure 5G). The clusters displayed distinct TF programs. Cluster 0 showed higher chromVAR deviation scores for ETS-family motifs, including ELK1, ETV6, and ELK3. Cluster 1 showed higher chromVAR deviation scores for EOMES- and TBR1-associated motifs, whereas cluster 2 exhibited a distinct NFIX-associated profile. We next investigated the chromatin regulatory landscape underlying these differences by comparing motif enrichment between grade 1 and grade 2 T cells (Figure 5H). In grade 2 tumors, we observed significant enrichment of TFE3 and CREM motifs. TFE3, in coordination with TFEB, modulates gene expression in T cells, particularly under stress conditions.^50^ CREM has been implicated in T cell tolerance and exhaustion,^51^ consistent with exhaustion-associated regulatory features in grade 2 T cells. In contrast, T cells in grade 1 tumors exhibited increased accessibility of POU family motifs, which have been linked to cell identity and stem-like regulatory programs.^52^ Collectively, these results suggest that T cells in grade 2 tumors are associated with more stress-responsive and exhaustion-associated regulatory features, whereas grade 1 T cells show chromatin features more consistent with quiescent or stem-like states. These grade-associated differences in T cell regulatory states may reflect distinct immune microenvironmental contexts across NF-PanNET grades.

### Chromatin landscape of the stromal microenvironment

CAFs (Cancer assoicate fibroblasts) are major components of the TME and have been implicated in tumor growth, matrix remodeling, and disease progression in various solid malignancies.^53^^,^^54^ Unsupervised clustering identified five distinct CAF subclusters (clusters 0–4), each exhibiting unique chromatin accessibility landscapes (Figures 6A and 6B). Gene activity scores of canonical CAF markers were computed to annotate these clusters (Figure 6C; Figure S5A). Clusters 0 and 1 were identified as classical myofibroblastic CAFs (myCAFs), characterized by elevated activity scores of ACTA2, TAGLN, FN1, and FAP-markers associated with contractility and electron cryomicroscopy (ECM) deposition, consistent with myofibroblastic features.^53^ Notably, pathway enrichment analysis indicated enrichment of EMT programs in CAFs from clusters 1 and 2 (Figure 6G). Cluster 3 displayed an immunomodulatory phenotype, with high activity of CD74 and THY1, suggestive of antigen-presenting CAFs (apCAFs) potentially involved in modulating immune responses.^55^ In contrast, cluster 2 exhibited a mechanobiological signature, marked by co-activation of DPT and TPM1, consistent with a matrix organization and contractility-associated state. Cluster 4 represented a matrix-remodeling subcluster, with elevated gene activity of MMP11 and the transcriptional regulator HOPX, suggesting a matrix-remodeling regulatory state potentially associated with invasive features.^56^ To understand the regulatory architecture underlying CAF diversity, we assessed both differential DACRs and chromVAR-derived TF deviation scores across subclusters (Figures 6D and 6E). Each cluster exhibited a unique set of DACRs, reflecting subcluster-specific regulatory programs. For instance, cluster 0 showed increased accessibility near metabolic genes such as PDK4, indicating a metabolic reprogramming signature. Cluster 3 exhibited open chromatin near DLL4, a Notch signaling ligand associated with angiogenic regulation.^57^ Cluster 4 showed accessible regions near genes related to cell motility and cytoskeletal remodeling, including CD164 and ARHGAP19, further supporting a motility- and cytoskeletal remodeling-associated state.

**Figure 6:**
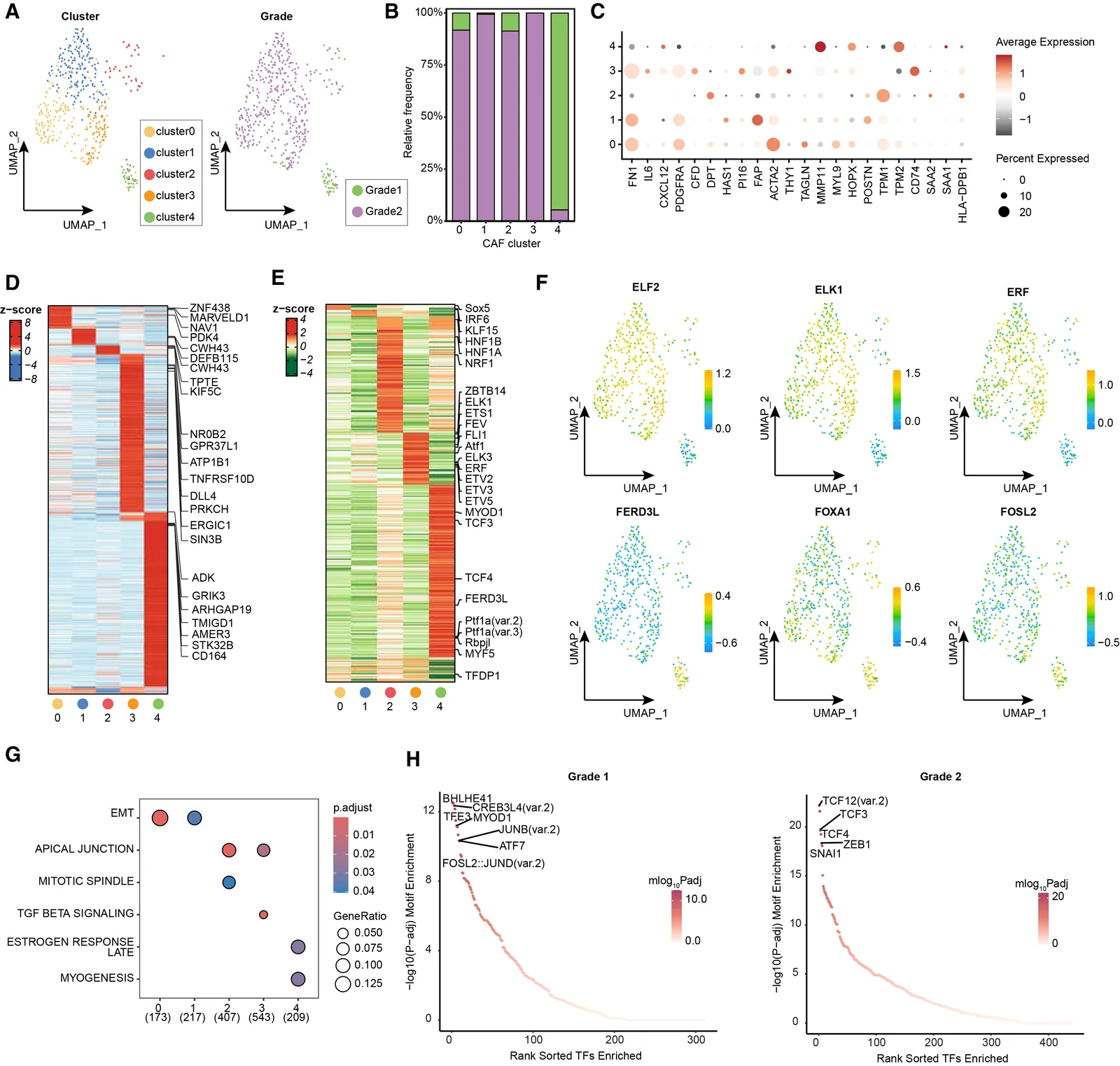
Chromatin landscape of the stromal microenvironment (A) UMAP projection of CAF snATAC-seq profiles showing annotated CAF subclusters (left) and tumor grade (right). (B) Stacked bar plot showing the relative grade composition of each CAF subcluster. (C) Dot plot showing gene activity scores of representative marker genes across CAF subclusters. Dot size represents the percentage of nuclei with detectable gene activity, and color indicates the average gene activity score. Dark red indicates higher gene activity scores, whereas dark gray indicates lower gene activity scores. (D) Heatmap of the top DACRs for each CAF subcluster. Color represents the z-scaled average normalized accessibility. (E) Heatmap of chromVAR-derived TF deviation scores across CAF subclusters. Color represents the z-scaled average chromVAR deviation score. (F) UMAP projections of selected TF deviation scores across CAFs. (G) Significant Hallmark pathway enrichments associated with DACRs showing increased accessibility in each CAF subcluster. Bubble size represents the gene ratio, and color indicates the adjusted *p* value. (H) TF motif enrichment within grade-associated CAF DACRs.

To further investigate grade-associated regulatory differences within CAFs, we performed motif enrichment analysis on grade-associated DACRs within CAFs. This revealed marked differences in motif-associated regulatory programs between tumor grades (Figure 6H). In grade 2, we observed strong enrichment of motifs for TFs associated with EMT and invasive programs, including SNAI1, ZEB1, and members of the TCF family (e.g., TCF4 and TCF12), all of which have been implicated in EMT and invasion. These findings indicate that grade 2 CAFs are associated with chromatin accessibility programs enriched for EMT- and invasion-related TF motifs. In contrast, grade 1 CAFs were enriched for motifs associated with the stress response, differentiation, and moderate proliferative programs, including FOSL2/JUNB (AP-1 complex), CREB3L1, and BHLHE41 (Figure 6H). Collectively, these results highlight substantial epigenetic heterogeneity within CAFs and identify grade-associated differences in EMT- and matrix-remodeling-related regulatory programs. These findings suggest that stromal chromatin states vary across NF-PanNET grades and may reflect broader differences in tissue remodeling within the TME.

### Spatial chromatin accessibility reveals grade-associated regulatory states in NF-PanNETs

Spatial ATAC-seq enables the mapping of chromatin accessibility within the native tissue architecture, complementing insights from snATAC-seq. We performed spatial ATAC-seq on tissues of 12 primary NF-PanNETs (Figures S6 and S7). H&E-stained tissue sections were annotated by pathologists (Figure S8). Integration of spatial ATAC-seq data from 12 NF-PanNET samples revealed six distinct spatial clusters defined by their chromatin accessibility profiles, along with 93,119 DACRs (Figure 7A; Figure S9A). Grade 1 and grade 2 tumors were predominantly represented by clusters c0–c4, whereas c5 was predominantly associated with the grade 3 tumor (Figure 7A, red circle and 7B). Cluster-specific *cis*-elements and their nearby genes were used to annotate the clusters, revealing a distinct chromatin accessibility signature in grade 3 tumors (Figure 7C). Mapping c5-associated spatial positions back onto the tissue section showed that they localized predominantly within the pathologist-annotated tumor region (Figure 7D; Figure S8L). Subsequently, we focused on grade 3 tumors and identified three major spatial clusters with tumor-, immune-, and fibroblast-associated chromatin accessibility profiles (Figure S10J). Motif enrichment analysis revealed overrepresentation of TCF3 and TCF12 motifs in sample PNET15 (Figure 7E). These transcription factors have been implicated in EMT, invasion, and stemness-related regulatory programs in several solid tumors, including pancreatic cancer, glioblastoma, and breast cancer.^58^^,^^59^ We further calculated gene activity scores and found that YAP1 showed significantly higher gene activity scores in grade 3 tumors than in grade 1 and grade 2 tumors (Figures 7F and 7H). YAP1, a downstream effector of the Hippo pathway, has established roles in tissue repair, regeneration, extracellular matrix remodeling, and tumorigenesis. Previous studies have linked elevated YAP1 activity to aggressive behavior and adverse clinical outcomes in pancreatic ductal adenocarcinoma.^60^ Our findings are consistent with and extend these observations to NF-PanNETs by showing increased YAP1-associated gene activity and chromatin accessibility in grade 3 tumors, suggesting that activation of the YAP-associated regulatory program may represent a feature of high-grade disease. Browser track visualization further showed increased chromatin accessibility at the YAP1 locus in grade 3 tumors (Figure 7G). Taken together, these findings indicate that grade 3 tumors exhibit a distinct chromatin accessibility landscape enriched for regulatory features previously associated with high-grade or aggressive tumor states.^34^

**Figure 7:**
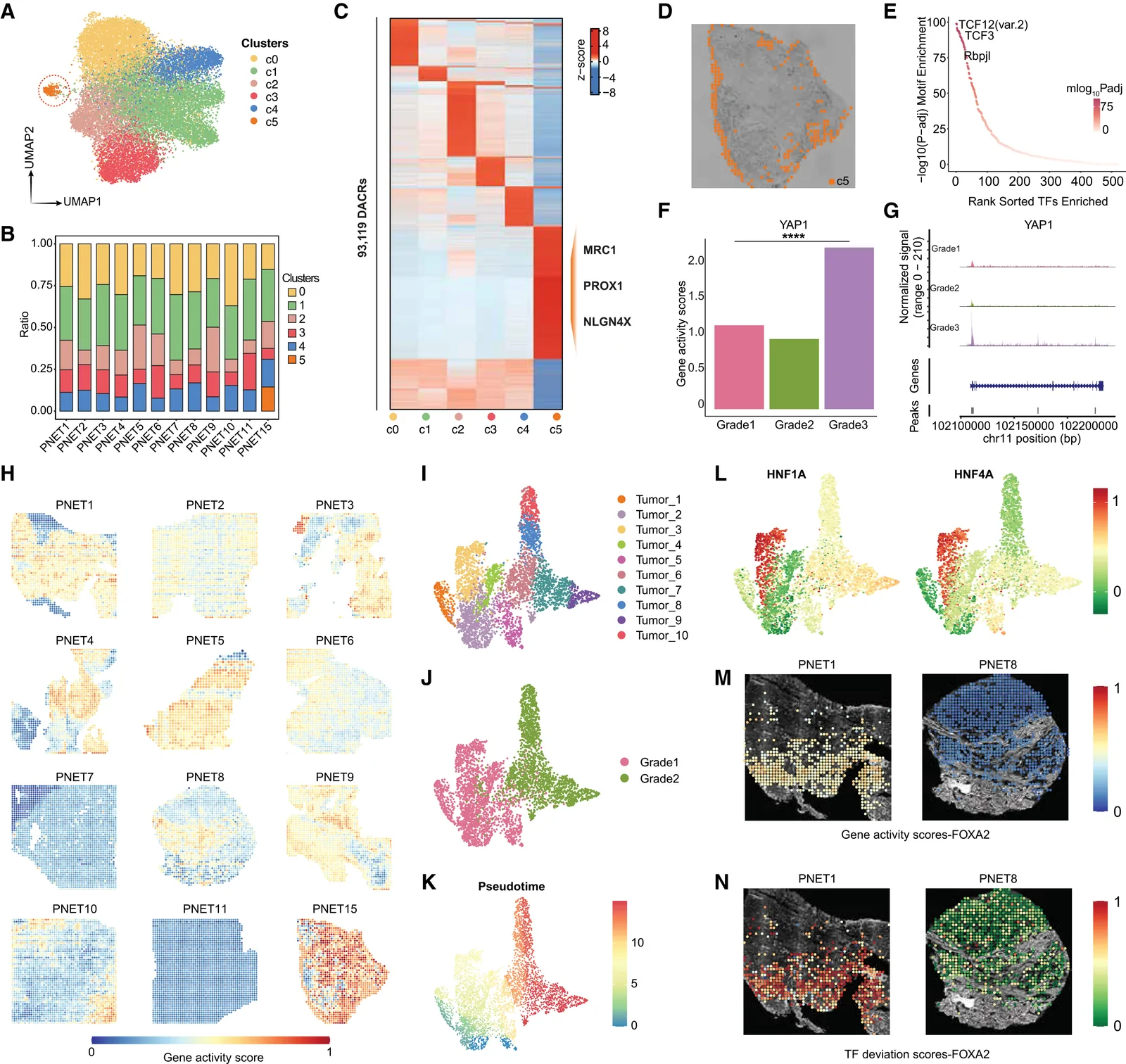
Spatial chromatin accessibility landscape across twelve NF-PanNETs (A) UMAP projection of spatial ATAC-seq profiles showing annotated spatial clusters. A red circle highlights cluster c5 associated with the grade 3 tumor. (B) Bar plot showing the relative proportion of each spatial cluster across individual samples. (C) Heatmap of top DACRs associated with the grade 3 spatial cluster. Color represents z-scaled average normalized accessibility across spatial clusters. (D) Spatial localization of cluster c5 within sample PNET5. (E) Enriched TF motifs identified in sample PNET15. (F) Bars represent the mean YAP1 gene activity score across spatial spots. Statistical significance was assessed using a two-tailed Mann-Whitney U test; ∗∗∗∗*p* < 0.0001. (G) Genome browser coverage plot showing chromatin accessibility at the YAP1 locus across tumor grades. (H) Spatial distribution of YAP1 gene activity scores across representative samples. (I and J) UMAP projections of tumor-associated spatial ATAC-seq profiles, colored by spatial subcluster (I) and tumor grade (J). (K) Pseudotime trajectory of grade-associated tumor chromatin states. (L) UMAP projections of chromVAR deviation scores for HNF1A and HNF4A motifs. (M) Spatial maps of FOXA2 gene activity scores in PNET1 and PNET8. (N) Spatial maps of FOXA2 chromVAR deviation scores in PNET1 and PNET8.

Next, we integrated tumor-associated spatial clusters from grade 1 and grade 2 NF-PanNETs and identified 10 subclusters, designated Tumor_1 to Tumor_10 (Figures 7I and 7J). Grade 1 tumors were predominantly represented by Tumor_1 to Tumor_5, whereas grade 2 tumors were enriched for Tumor_6 to Tumor_10. ChromVAR deviation scores for HNF1A, HNF4A, and HNF4G motifs were higher in grade 1 tumors, consistent with the patterns observed in the snATAC-seq analysis (Figure 7L; Figures S9B and S9C). To examine whether grade 1- and grade 2-associated spatial tumor states could be organized along a continuous chromatin accessibility trajectory, we performed pseudotime inference using the spatial ATAC-seq profiles. Grade 1-associated tumor spots were enriched toward one end of the inferred trajectory, whereas grade 2-associated tumor spots were enriched toward the opposite end, indicating a continuum of grade-associated chromatin accessibility states (Figure 7K). This pattern was consistent with the grade-associated continuum observed in the snATAC-seq dataset (Figure 3A). Consistent with this observation, FOXA2 showed higher chromVAR deviation scores toward the grade 1-associated end of the snATAC-seq trajectory, and both FOXA2 chromVAR deviation scores and gene activity scores were higher in PNET1 (grade 1) than in PNET8 (grade 2) in the spatial ATAC-seq dataset (Figures 7M and 7N). These findings further support the concordance of grade-associated FOXA2 regulatory patterns between the snATAC-seq and spatial ATAC-seq datasets.

### Spatial association between tumor accessibility states and myCAF-associated regions in NF-PanNETs

Previous studies have reported that patients with a high stromal proportion tend to have a worse prognosis, particularly when the stroma exhibits an infiltrative growth pattern, where stromal and tumor cells intermix without a clear boundary.^61^ Among the stromal components, myCAFs, commonly characterized by FAP and ACTA2 expression, have been reported to localize near tumor cells and to be associated with EMT, matrix remodeling, and tumor-promoting stromal states. We identified myCAFs in the snATAC-seq datasets and sought to investigate the spatial relationship between tumor-associated and fibroblast-associated accessibility states using spatial ATAC-seq. After dimensionality reduction and clustering, spatial clusters were annotated based on marker gene activity and pathway enrichment analyses (Figures S10A–S10I). Interestingly, two distinct tumor-associated accessibility states, designated Tumor1 and Tumor2, were identified in sample PNET8 (Figures 8A–8C). Spatial ATAC-seq analysis revealed that both Tumor1-and Tumor2-associated spatial spots were located near fibroblast-associated regions, with Tumor2 being notably adjacent to regions enriched for high gene activity scores of ACTA2 and FAP, consistent with spatial proximity to myCAF-associated regions (Figures 8D and 8E). To quantify this observation, we calculated the spatial distances between myCAF-associated regions and the two tumor-associated accessibility states, showing that Tumor2-associated spots were spatially closer to myCAF-associated regions (Figure 8F). Pathway enrichment analysis further revealed that Tumor2 was enriched in proliferative signaling pathways, such as E2F targets, MYC targets, and mTORC1 signaling, alongside oxidative phosphorylation. In contrast, Tumor1 exhibited enrichment of genes involved in the KRAS signaling pathway (Figure 8C).

**Figure 8:**
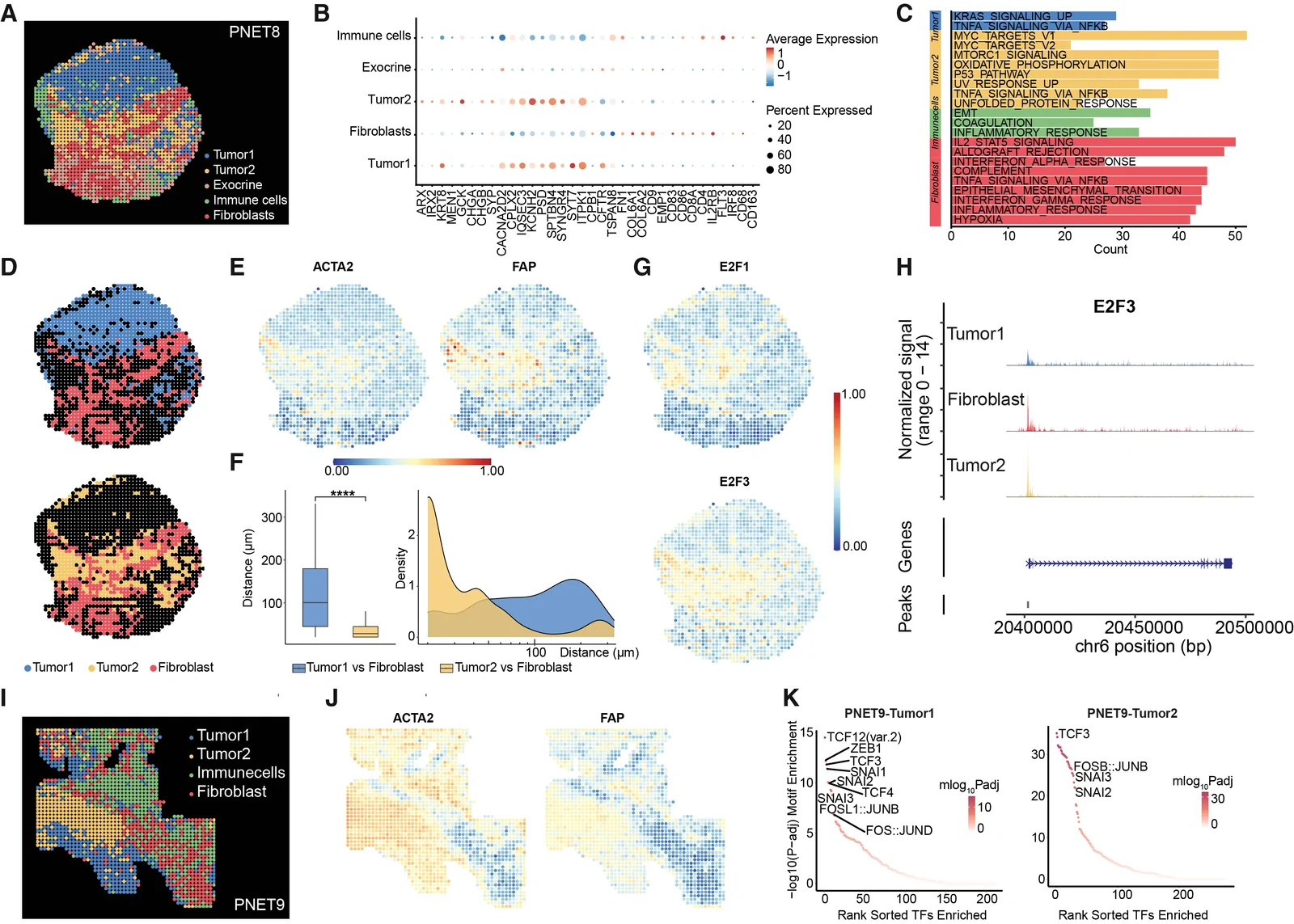
Intra- and intertumoral heterogeneity revealed by spatial chromatin accessibility (A) Spatial distribution of annotated spatial clusters in PNET8. (B) Dot plot showing gene activity scores of representative marker genes across PNET8 spatial subclusters. Dot size indicates the proportion of spots with gene activity; color intensity reflects the average gene activity score. Dark red indicates higher gene activity scores, dark blue indicates lower gene activity scores. (C) Enriched hallmark pathways associated with DACRs showing increased accessibility in each PNET8 spatial subcluster. (D) Unsupervised clustering and spatial localization of Tumor1-, Tumor2-, and fibroblast-associated spatial clusters in PNET8. (E) Spatial gene activity maps of ACTA2 and FAP in PNET8. (F) Distribution of nearest spatial distances from Tumor1- and Tumor2-associated spots to fibroblast-associated spots in PNET8. Statistical significance was assessed using a two-tailed Mann-Whitney U test (∗∗∗∗*p* < 0.0001). (G) Spatial gene activity maps of E2F1 and E2F3 in PNET8. (H) Chromatin accessibility profiles at the E2F3 locus across spatial subclusters. (I) Spatial distribution of annotated spatial clusters in sample PNET9. (J) Spatial gene activity maps of ACTA2 and FAP in PNET9. (K) Enriched TF motifs in Tumor1- and Tumor2-associated spatial subclusters of PNET9.

We extended this spatial analysis to other samples (Figures S11A–S11C). Tumor-associated regions in PNET1, PNET4, and PNET6 were located near spatial regions with high ACTA2 and FAP gene activity and were enriched in proliferative pathways, similar to Tumor2 in PNET8. In contrast, tumor-associated regions in PNET7 and PNET10 were spatially associated with lower ACTA2 and FAP gene activity and were enriched in the KRAS pathway. Notably, intra-tumor heterogeneity was also observed in PNET9, where two distinct tumor-associated accessibility states were identified (Figures 8I and 8J; Figure S11D). As in PNET8, Tumor2 in PNET9 was spatially associated with myCAF-associated regions and enriched in proliferative pathways, while Tumor1 was characterized by KRAS pathway activity. In summary, tumor-associated regions spatially proximal to myCAF-associated regions tended to show stronger enrichment of proliferation-associated regulatory programs, whereas more spatially distant tumor-associated regions showed greater enrichment of KRAS-related programs. Fibroblast-associated regions proximal to tumor-associated regions also showed enrichment of EMT- and hypoxia-related programs. Further analysis revealed that E2F1 and E2F3 showed higher gene activity scores in Tumor2 than in Tumor1 (Figure 8G). Browser track visualization of E2F3 showed elevated chromatin accessibility in Tumor2 (Figure 8H), consistent with the proliferation-associated program identified in Tumor2. Additionally, motif enrichment analysis across samples showed that FOX motifs were enriched in highly proliferative tumors, consistent with their role in regulating the cell cycle (Figure S11E). In contrast, tumor-associated regions with stronger KRAS pathway enrichment showed increased SNAI1, SNAI2, and SNAI3 motif enrichment (Figure 8K; Figures S11F and S11G). KRAS activation can induce SNAI1 and SNAI2 expression, which repress epithelial gene expression and promote EMT.^62^^,^^63^ These motif patterns are consistent with previously described links between KRAS-associated signaling, SNAI-family regulatory programs, EMT, and invasive tumor phenotypes. Together, these findings highlight both intertumoral and intratumoral heterogeneity in spatial chromatin accessibility, as exemplified by PNET8 and PNET9, where distinct tumor-associated accessibility states were observed within different stromal contexts. Tumor-associated regions proximal to myCAF-associated regions showed stronger enrichment of proliferation-related regulatory programs, whereas spatially more distant regions showed greater enrichment of KRAS-associated programs. These observations identify spatial associations between tumor regulatory states and fibroblast-associated regions and highlight the utility of spatial ATAC-seq for resolving local regulatory heterogeneity within NF-PanNET tissues.

## Discussion

Our integrative analysis of snATAC-seq and spatial ATAC-seq provides a high-resolution view of the chromatin regulatory landscape of NF-PanNETs. By profiling chromatin accessibility at single-nucleus resolution and within native tissue architecture, we identify grade-associated regulatory states across tumor, immune, and stromal compartments and further examine how these states are organized within distinct spatial contexts.

Recent single-cell transcriptomic studies of PanNETs have revealed substantial tumor and microenvironmental heterogeneity associated with histological grade, genomic instability, and metastatic state. Our findings extend this transcriptomic framework by showing that NF-PanNET heterogeneity is also reflected in chromatin accessibility, *cis*-regulatory element usage, and TF-associated regulatory activity. We identified multiple chromatin-defined tumor states within the same histological grade, including HNF1A/FOXA2-associated programs that were more prominent in lower-grade tumors and E2F-, AP-1-, SNAI-, and YAP/TEAD-associated programs that were more prominent in higher-grade tumors, together with heterogeneous myeloid, T cell, and fibroblast regulatory states. Differences in cellular resolution between our study and previous transcriptomic studies likely reflect differences in assay modality, cohort composition, and sampling depth. Thus, our work provides a complementary regulatory and spatial perspective on the cellular heterogeneity previously described by single-cell transcriptomic profiling rather than a direct replication of previously defined cellular taxonomies.

Across tumor cells, we observed marked grade-associated differences in TF-linked chromatin programs. Grade 1 tumors showed higher activity of lineage-associated factors such as HNF1A, HNF4A, and FOXA2, whereas grade 2 tumors showed greater activity of proliferative and stress-responsive regulators, including members of the E2F and AP-1 families. Grade 3 tumors additionally showed increased YAP1 gene activity and accessibility at the YAP1 locus, together with enrichment of regulatory programs previously associated with EMT, stemness, and aggressive tumor phenotypes in other malignancies.^60^^,^^64^ These findings support the presence of distinct regulatory states across NF-PanNET grades. Importantly, however, our samples were collected cross-sectionally, and the pseudotime analyses therefore represent inferred continua of chromatin states rather than direct evidence of a chronological transition from grade 1 to grade 2 or grade 3 disease. The spatial ATAC-seq analysis further revealed that tumor-associated chromatin states were organized within distinct stromal contexts. In representative tumors such as PNET8 and PNET9, tumor-associated regions located near fibroblast- and myCAF-associated regions showed stronger enrichment of proliferation-related programs, including E2F, MYC, and mTORC1 signaling, whereas more spatially distant tumor-associated regions showed greater enrichment of KRAS- and SNAI-associated regulatory programs. Fibroblast-associated regions proximal to tumor-associated regions also showed EMT- and hypoxia-related pathway enrichment. These spatial relationships identify local coupling between tumor and stromal regulatory states but do not establish a causal direction of interaction. In particular, the myCAF-associated patterns may reflect tumor-induced reactive stroma or broader tissue remodeling, rather than demonstrating that fibroblasts directly induce the associated tumor regulatory programs. Furthermore, because the spatial ATAC-seq measurements represent multicellular spatial positions rather than single cells, gene activity and pathway scores may reflect both cellular composition and cell-state variation within individual spots.

Our study provides a chromatin-level and spatial framework for understanding regulatory heterogeneity in NF-PanNETs. The recurrent grade-associated patterns involving HNF/FOXA2, E2F/AP-1, YAP/TEAD, and SNAI-associated programs provide hypotheses for future mechanistic investigation, while the spatial association between tumor accessibility states and fibroblast-rich regions highlights the importance of considering local tissue context when interpreting tumor regulatory states.

### Limitations of the study

Several limitations of the present study should be acknowledged. First, the cohort size was modest, with particularly limited representation of grade 3 tumors, which constrains the generalizability of grade-associated patterns. Second, the spatial ATAC-seq measurements were analyzed at the spot level, and formal spatial deconvolution was not performed because the sparsity of the data and the similarity of related tumor and stromal states made robust estimation of quantitative cell-type fractions challenging. Accordingly, the spatial analyses were based on clustering, gene activity, regulatory signatures, and spatial proximity rather than inferred cell proportions. Third, although the integration of snATAC-seq and spatial ATAC-seq identified reproducible grade-associated and spatially organized regulatory programs, these findings have not yet been independently validated using orthogonal approaches such as multiplex immunofluorescence, spatial transcriptomics, or functional perturbation. The candidate regulatory programs identified here should therefore be regarded as discovery-oriented and hypothesis-generating. Future studies integrating chromatin accessibility with orthogonal spatial and functional assays, larger independent cohorts, and longitudinal clinical data will be important for establishing the biological, mechanistic, and clinical relevance of these regulatory states.

## Resource availability

### Lead contact

Requests for further information, resources, and reagents should be directed to and will be fulfilled by the lead contact, Yang Liu (yang.liu.yl2224@yale.edu).

### Materials availability

This study did not generate new unique reagents.

### Data and code availability

• The snATAC-seq and spatial ATAC-seq datasets generated in this study are publicly available through the NCBI Gene Expression Omnibus (GEO) under accession number GSE304813, which is also listed in the key resources table. Detailed quality-control metrics, annotated CRE information, TF activity data, and numerical source data underlying the indicated figures are provided in Data S1.
• All custom scripts used for data preprocessing, analysis, and visualization are freely available at https://github.com/Xiangjun99/NF-PanNETs-ATAC-project and are listed in the key resources table.
• Any additional information required to reanalyze the data reported in this paper is available from the lead contact upon request.

## Acknowledgments

We acknowledge the support from Department of Pathology and Department of Neurology at 10.13039/100007184Yale School of Medicine. We also thank the Yale Pathology Tissue Services. The research is supported by Maximizing Investigators Research Award (MIRA) for Early Stage Investigators R35 GM150838 and 10.13039/100000002NIH R01 HL173271 (to Y.L.). The research is also supported, in part, by grant no. #IRG-21-132-60-IRG from the 10.13039/100000048American Cancer Society. We also acknowledge the support of Leslie H. Warner Postdoctoral Fellowship to D.W.

## Author contributions

Conceptualization, Y. Liu., J.D., and D.W.; methodology, D.W., X.D., Y. Liu., F.G., G.L., J.D., and Y.J.; investigation, D.W., X.D., and Y. Liu.; visualization, X.D. and Y. Liu.; writing – original draft, X.D. and D.W.; writing – review and editing, X.D., D.W., and Y. Liu.; resources, F.G., G.L., L.L., S.H., D.Z., Y. Liang, J.X., Y.J., J.L., P.K., and J.K.; supervision, J.D. and Y. Liu.; funding acquisition, J.D. and Y. Liu.

## Declaration of interests

The authors declare no competing interests.

## STAR★Methods

### Key resources table

**Table:** 

REAGENT or RESOURCE	SOURCE	IDENTIFIER
**Biological samples**		

Human primary pancreatic neuroendocrine tumor (NF-PanNET) specimens	Yale University School of Medicine	Yale HIC no. 1203009817

**Chemicals, peptides, and recombinant proteins**		

pA-Tn5 Transposase – unloaded	C01070010-20	Diagenode
Tagmentation Buffer (2x)	C01019043-5000	Diagenode
Digitonin	G9441	Promega
5M NaCl	J60434.AK	Thermo Fisher Scientific
Paraformaldehyde solution (16%)	043368.9M	Thermo Fisher Scientific
Glycine	50046	Sigma-Aldrich
Proteinase K	EO0491	Thermo Fisher Scientific
EDTA (0.5 M)	AM9260G	Thermo Fisher Scientific
Fisher Healthcare Tissue-Plus™ O.C.T. Compound	23-730-571	Fisher Scientific
Triton X-100	T8787	Sigma-Aldrich
T4 DNA Ligase	M0202L	New England Biolabs
T4 DNA Ligase Reaction Buffer	B0202S	New England Biolabs
NEBNext High-Fidelity 2X PCR Master Mix	M0541L	New England Biolabs
DPBS	14190144	Thermo Fisher Scientific
DNA Clean & Concentrator-5	D4014	Zymo Research
dNTP mix	R0192	Thermo Fisher Scientific
EvaGreen Dye 20X in Water	31000-T	Biotium
SPRIselect beads	B23318	Beckman coulter
Poly-L-Lysine Slide	63478-AS	Election microscopy sciences
D5000 ScreenTape	5067-5588	Agilent
D5000 reagents	5067-5589	Agilent
Silicon wafer	C04004	WaferPro
Photoresist SU-8 2010	SU-8 2010	Microchem Laboratory
Tween 20™ 10%	11332465001	Sigma-Aldrich
Tris-HCl, 1M Solution, pH 8.0	J22638.K2	Thermo Fisher Scientific
UltraPure™ DNase/RNase-Free Distilled Water	10977015	Thermo Fisher Scientific
DAPI	40043	Biotium
Primers, ligation linkers, DNA barcodes	IDT	See Table S1
Chromium Nuclei Isolation kit	1000493	10X Genomics

**Deposited data**		

snATAC-seq data generated in this study	This paper	GSE304813
Spatial ATAC-seq data generated in this study	This paper	GSE304813
Analysis code	This paper	https://github.com/Xiangjun99/NF-PanNETs-ATAC-project

**Software and algorithms**		

Cell Ranger ATAC	10x Genomics	v2.0
R	R for Statistical Computing	v4.3.2
Signac	Stuart et al.^65^	v1.12.0
Seurat	Hao et al.	v5.0.1
Harmony	Korsunsky et al.	v1.2.0
chromVAR	Schep et al.^31^	v1.24.0-
chromVARmotifs	Bioconductor/authors	v0.2.0
clustree	Zappia and Oshlack	v0.5.1
ChIPpeakAnno	Zhu et al.	v3.36.0
clusterProfiler	Wu et al.	v4.10.0
ArchR	Granja et al.^66^	v1.0.1
ggplot2	Wickham et al.	v3.5.1
MATLAB	MathWorks	R2020b

**Other**		

Human reference genome	UCSC/Genome Reference Consortium	GRCh38/hg38

### Experimental model and study participant details

#### Clinical specimens and ethics statements

Human primary pancreatic neuroendocrine tumor (NF-PanNET) specimens were obtained from 15 treatment-naïve patients undergoing surgical resection at Yale School of Medicine. Samples were verified by standard pathology and classified according to histopathologic tumor grade, including six Grade 1, eight Grade 2, and one Grade 3 tumors. Nine specimens were analyzed by snATAC-seq and 12 specimens were analyzed by spatial ATAC-seq, with six specimens profiled using both modalities. Demographic and clinical characteristics of the participants, including age, gender, Hispanic/Latina ethnicity, and tumor grade, are provided in Table S4.

All human samples were collected with informed consent in accordance with the Yale University Institutional Review Board (IRB) at the Yale University School of Medicine (Yale HIC approval number: 1203009817). Tissue collection and OCT embedment were conducted under Yale University IRB approval and oversight by the Tissue Resource Oversight Committee. Written informed consent for participation was obtained in accordance with the Declaration of Helsinki. Each sample was handled in compliance with HIPAA regulations, University Research Policies, Pathology Department diagnostic requirements, and Hospital by-laws. Freshly resected tissues were embedded in OCT and snap-frozen in liquid nitrogen before subsequent processing.

Given the modest cohort size and unequal representation of tumor grades, the study was not powered to assess sex- or gender-associated differences in chromatin accessibility. Therefore, the influence of sex or gender on the reported findings could not be determined.

### Method details

#### Tissue section preparation and staining

The freshly dissected human samples were immersed into OCT and snapped frozen with liquid nitrogen. Before sectioning, the frozen tissue block was warmed to the temperature of cryotome cryostat (−20 °C). Tissue block was then sectioned into thickness of 7–10 μm and placed in the center of a poly-L-lysine coated glass slide (electron microscopy sciences, CatLog no. 63478-AS). Serial tissue sections were collected simultaneously for DBiT-ATAC-seq and H&E staining.

The adjacent sections underwent a standard H&E staining procedure. All staining is performed at Yale University School of Medicine, Department of Pathology and at YPTS. These procedures adhered to Clinical Laboratory Improvement Amendments (CLIA)-certified laboratory protocols as well as YPTS’s rigorous standard protocols, ensuring precision and accuracy in the analysis of tissue samples.

#### Single-nucleus ATAC (snATAC) assay

OCT-embedded tissues were sectioned to generated 5–10 50 μm scrolls using a microtome (Finesse ME+, Thermo Scientific, UK). Single cell suspensions were generated from the scrolls using10X Chromium Nuclei Isolation Kit (1000493, 10x Genomics, US) according to the manufacturer’s recommendations. Nucleus suspensions were then flow-sorted with the BD FACSMelody, where 7AAD intensity was used to gate the desired populations.

The snATAC-seq libraries were prepared using the Chromium Next GEM Single Cell ATAC Reagent kits (1000390, 10x Genomics, US) according to the standard protocol. Initially, the isolated nucleus suspensions were incubated in a transposition mix containing a transposase enzyme, facilitating preferential fragmentation of DNA in open chromatin regions and introducing adapter sequences. Subsequently, approximately 10,000 transposed nuclei were loaded onto the Chromium Next Chip H. During GEM generation, gel beads introduced a spacer sequence facilitating barcode attachment to transposed DNA fragments allowing individual nuclei to be uniquely labeled during subsequent amplification. After GEM incubation, DNA fragments were purified following the standard protocol, and the purified DNA fragments were amplified via PCR to generate sufficient material for sequencing. Following quality assessment, the snATAC library underwent paired-end 150 bp read sequencing on the Illumina NovaSeq 6000 sequencing system.

#### Spatial ATAC-seq

DNA oligos including barcodes, barcodes linkers, indexes and transposome oligos are all procured from integrated DNA Technologies (IDT, Coralvile, IA) and the sequences were listed in Tables S1–S3. The transposome was assembled beforehand following the manufacturer’s guidelines using unloaded Tn5 transposase (Diagenode, no. C01070020). OCT-embedded tissue sections were taken from the −80 °C freezer and allowed to return to room temperature for 5–10 min. The sections were then fixed with 0.2% formaldehyde in DPBS for 5 min and quenched with 1.25 M glycine for an additional 5 min. After fixation, the tissue was washed twice with 1 mL of DPBS, and the fixed tissues were then permeabilized for 20 min at room temperature with freshly made permeabilization buffer (10 mM Tris-HCl, pH 7.4, 10 mM NaCl, 3 mM MgCl2, 0.01% Tween 20, 0.01% NP-40, 0.001% digitonin, 1% BSA). 1 mL wash buffer (10 mM Tris-HCl, pH 7.4, 10 mM NaCl, 3 mM MgCl2, 0.01% Tween 20, 1% BSA) was used to wash the tissue twice after permeabilization. The tissues were then quickly rinsed with deionized water, air-dried and installed with PDMS reservoir and clamps for tagmentation. For each sample, 100ul transposition mix containing 50 μL 2× tagmentation buffer, 33 μL 1× DPBS, 1 μL 10% Tween 20, 1 μL 1% digitonin, 5 μL transposome, 10 μL nuclease-free H2O was added followed by incubation at 37 °C for 30 min. After tagmenetation, 100 μL 40 mM EDTA was added to the tissues instantly to stop the transposition. Finally, the EDTA was removed, and tissues were washed with DPBS for three times, rinsed with water and air-dried for imaing. Spatial barcodes are made days before the tissue processing. Barcode (100 μM) and ligation linker (100 μM) were annealed at a 1:1 ratio in 2X annealing buffer (20 mM Tris-HCl pH 8.0, 100 mM NaCl, 2 mM EDTA) with the following PCR program: 95 °C for 5 min, slow cooling to 20 °C at a rate of −0.1 °C/s, followed by 12 °C for 3 min. Barcode ligation was performed through three consecutive procedures: whole image capture, first PDMS device attachment, imaging, loading barcode A (A1-A50) and second PDMS device attachment, imaging, loading barcode B (B1-B50). For downstream alignment and analysis, tissues were scanned three times using a 10× objective on the EVOS M7000 Imaging System. The first image was taken before placing the first PDMS device, covering the entire tissue area, while the second and third images, covering only the ROI, were taken after attaching the first and second PDMS devices, respectively. Microfluidic barcoding was performed by positioning the PDMS device channels over the ROI, with the PDMS device and tissue slide clamped tightly using a homemade acrylic clamp. The ligation mix for barcodes A and B was prepared with 69.5 μL of RNase-free water, 27 μL of T4 DNA ligase buffer (10X, New England Biolabs), 11 μL T4 DNA ligase (400 U/μL, New England Biolabs), and 5.4 μL of Triton X-100 (5%). A 5 μL ligation mix containing 4 μL of the ligation mix and 1 μL of 25 μM DNA barcode A (A1-A50) or barcode B (B1-B50) was added to each of the 50 channels’ inlets and pulled in using a house vacuum for less than 3 min. The reaction occurred at room temperature for 30 min in a wet box. After reaction, the clamp was removed, the PDMS was carefully peeled off, and the slide was washed for 5 min in a Falcon tube containing 50 mL DPBS and air-dried for the next barcoding or lysis. After the second barcoding, a square well PDMS gasket was placed over the ROI where both barcodes had flowed. The reservoir created by the PDMS gasket was loaded with 50–100 μL of freshly made lysis buffer (0.4 mg/mL proteinase K, 1 mM EDTA, 50 mM Tris-HCl pH 8.0, 200 mM NaCl, 1% SDS), depending on the reservoir size. The slides were incubated in a wet box at 55 °C for 2 h. The lysates in the reservoir were then collected in a 1.5 mL DNA low-binding tube and incubated at 55 °C overnight. Genomic fragments in the lysate were purified using the Zymo DNA Clean & Concentrator-5 and eluted into 22 μL of RNase-free water. The ATAC library was built following a direct PCR. The PCR mix, consisting of 20 μL DNA solution, 25 μL of 2X NEBNext Master Mix, 2.5 μL of 25 μM indexed i7 primer, 2.5 μL of 25 μM P5 PCR primer, and 2.5 μL 20X EvaGreen, was run with the following program in a qPCR machine: 72 °C for 5 min, 98 °C for 30 s, followed by cycles of 98 °C for 10 s, 63 °C for 30 s, and 72 °C for 1 min. The reaction was stopped when the signal began to plateau. The PCR product was purified using SPRIselect beads at a 1:1 ratio and eluted into 20 μL of nuclease-free water. Finally, the library quality was checked using Agilent Bioanalyzer High Sensitivity Chips, and next-generation sequencing (NGS) was conducted on an Illumina NovaSeq 6000 sequencer (paired-end, 150-base-pair mode).

#### snATAC data processing

Single-nucleus ATAC-seq (snATAC-seq) data were processed using Cell Ranger ATAC (v2.0, 10x Genomics, https://support.10xgenomics.com), including read alignment, removal of PCR duplicates, peak calling, and generation of fragment counts in a cell-by-peak matrix for each sample. Prior to merging, a unified peak set was constructed by aggregating peaks from all samples, followed by quantification in each dataset. For downstream analysis, the filtered_peak_bc_matrix.h5, singlecell.csv, and fragments.tsv.gz files were imported into R v4.3.2^65^ and analyzed using Signac v1.12.0 and Seurat v5.0.1. A ChromatinAssay was constructed from the Cell Ranger ATAC peak-by-cell count matrix and corresponding fragment file. Gene annotations were obtained from the EnsDb.Hsapiens.v98 annotation package. Chromosome naming conventions were converted to the UCSC style and assigned to the hg38 genome build. Quality-control metrics were calculated using Signac, and nuclei with fewer than 500 unique fragments or a transcription start site enrichment score below 3 were excluded from downstream analysis. To correct for batch effects across samples, the Harmony algorithm was applied with default parameters. For dimensionality reduction, the peak-by-cell matrix was normalized using term frequency–inverse document frequency transformation with RunTFIDF. Informative features were selected using FindTopFeatures with min.cutoff = “q0”, followed by latent semantic indexing using RunSVD. The first LSI component was excluded because it was correlated with sequencing depth. LSI dimensions 2–10 were used for construction of the nearest-neighbor graph with FindNeighbors and for generation of the UMAP embedding with RunUMAP. Cell clusters were identified using the Louvain community-detection algorithm implemented in Seurat’s FindClusters function, with algorithm = 1. For each major cell compartment, clustering resolutions ranging from 0.1 to 1.0 were evaluated and compared using clustree (Figure S12). The final resolution was selected based on the persistence of cluster structure across adjacent resolutions, separation in the UMAP embedding, consistency across samples, accessibility of canonical lineage markers, and avoidance of over-fragmentation of biologically coherent populations. The selected resolutions were 0.6 for tumor cells, 0.3 for the overall immune-cell compartment, 0.4 for T cells, 0.8 for myeloid cells, and 0.4 for cancer-associated fibroblasts. Unless otherwise specified, all other parameters were set to their package defaults.

#### Spatial ATAC-seq data preprocessing

Spatial ATAC-seq data were preprocessed using a previously published pipeline (https://github.com/dyxmvp/Spatial_ATAC-seq). Briefly, Read 1 was filtered using Linker 1 and Linker 2. The filtered sequences were then reformatted into the Cell Ranger ATAC (10x Genomics) input format. In the new format, genomic sequences were retained in Read 1, while Barcode A and Barcode B were incorporated into Read 2. The resulting FASTQ files were aligned to the human reference genome (GRCh38), followed by duplicate removal and fragment counting using Cell Ranger ATAC v2.0. Fragment files were generated for downstream analysis, with each entry containing genomic coordinates and the corresponding spatial barcode information (Barcode A × Barcode B).

#### Spatial ATAC-seq data processing and integration

Spatial ATAC-seq count matrices generated by Cell Ranger ATAC were processed separately for each sample using Signac and Seurat. The raw peak-by-barcode matrix was imported with Read10X_h5. Spatial barcodes were converted to their corresponding two-dimensional coordinates using a predefined barcode lookup table. Barcodes located outside the tissue region, as determined from the sample-specific spatial coordinate file, were removed before downstream analysis. A ChromatinAssay was then created from the filtered peak-by-barcode matrix and corresponding fragments.tsv.gz file using CreateChromatinAssay with min.cells = 1 and min.features = 10. Peaks located on non-standard chromosomes were excluded. Gene annotations were obtained from EnsDb.Hsapiens.v98, converted to UCSC chromosome naming conventions, assigned to the hg38 genome build, and added to the chromatin assay.

For each sample, chromatin accessibility data were normalized using term frequency–inverse document frequency transformation with RunTFIDF. Features were retained using FindTopFeatures(min.cutoff = “q0”), followed by latent semantic indexing with RunSVD. The first LSI component was excluded because of its association with sequencing depth, and LSI dimensions 2–10 were used for UMAP visualization and nearest-neighbor graph construction with RunUMAP and FindNeighbors, respectively. Initial within-sample clustering was performed using FindClusters with the smart local moving algorithm (algorithm = 3) and resolution = 0.4. Cluster identities were assigned by examining gene activity scores for canonical tumor, exocrine, fibroblast, and immune-lineage markers. Gene activity matrices were generated using GeneActivity, added to each Seurat object as an RNA assay, and normalized using LogNormalize. Cluster assignments were subsequently mapped back to their spatial coordinates using the barcode lookup table.

For cross-sample analysis, the 12 individually processed spatial ATAC-seq objects were merged after assigning sample and histological-grade metadata. The combined peak matrix was reprocessed using RunTFIDF, FindTopFeatures(min.cutoff = 20), and RunSVD. Batch-associated variation across samples was corrected using Harmony on the LSI embeddings, with sample identity specified as the grouping variable. The Harmony-corrected embeddings were used to construct the nearest-neighbor graph with FindNeighbors using dimensions 2–25 and n.trees = 100. Louvain clustering was performed using FindClusters with algorithm = 1, and UMAP coordinates were generated from the Harmony embeddings using dimensions 2–25. The resulting clusters were evaluated according to sample composition, tumor grade, gene activity of canonical lineage markers, and their spatial distribution within the tissue sections.

#### Gene activity and TF dynamics analysis

Gene activity scores were calculated using Cicero,^67^ which aggregates accessibility signals across gene promoters and their *cis*-co-accessible distal regulatory elements. TF activity was assessed using the RunChromVAR function in Signac, based on position weight matrices (PWMs) from the chromVARmotifs R package.^31^ Per-cell motif deviation scores were used to infer relative TF activity across cell types. Differentially accessible chromatin regions (DACRs) and TF motifs were identified using the FindAllMarkers function (min.pct = 0.1, logfc.threshold = 0.25).

#### Cell cluster annotation

Cell clusters were annotated by comparing gene activity scores of known marker genes. Tumor cells were defined by expression of ARX, IRX2, KRT8, MEN1, GCK, CHGA, CHGB, SYP, CACNA2D2, CPLX2, IQSEC3, KCNH2, PSD, SPTBN4, SYNGR4, SYT7, and ITPK1. Exocrine cells were marked by CPB1, CFTR, and TSPAN8. Fibroblasts were identified via FN1, COL6A1, COL6A2, CD9, and EMP1. Immune cell types were annotated as follows: B cells (CD83, CD86, CD19, MS4A1, CD79A, CD79B), T cells (CD8A, CD8B, CD4), NK cells (IL2RB, SPON2, KLRD1, CD160), conventional dendritic cells (cDCs; FLT3, IRF8), and macrophages (CD68, CD163, LAMP2, ITGAM, LGALS3). CAF subtypes were defined as follows: inflammatory CAFs (iCAFs; IL6, CXCL12), myofibroblastic CAFs (myCAFs; FAP, ACTA2), and antigen-presenting CAFs (apCAFs; CD74, HLA-DPB1, HLA-DRA, HLA-DRB1).

#### Identification and annotation of cell-type specific chromatin accessible regions

Cell type–specific ACRs (peaks) were identified using the FindAllMarkers function in Seurat, with the following thresholds: log fold change >0.5, minimum of 50 cells per group, and adjusted *p* < 0.05. Peaks were annotated to the nearest genes using the annotatePeak function in the ChIPseeker R package, with promoter regions defined as ±3 kb from the transcription start site (TSS). Chromatin states were annotated using the core 15-state model from the NIH Roadmap Epigenomics Mapping Consortium (https://egg2.wustl.edu/roadmap/web_portal/chr_state_learning.html#core_15state). ChromHMM states were assigned to peak regions using the annotatedPeak function from ChIPpeakAnno with default parameters.

#### Motif enrichment analysis

Motif enrichment was performed using a hypergeometric test implemented in the FindMotifs function of Signac, comparing the frequency of TF motifs in a target peak set versus background peaks matched for GC content.

#### TF footprinting analysis

TF footprinting analysis was conducted using Signac. Motif positions were first added to the peak matrix. The Footprint function was used to compute the normalized observed/expected Tn5 insertion frequency surrounding each motif instance across the genome. The resulting footprints were visualized using the PlotFootprint function.

#### Pseudotime analysis

To assess gene and TF activation dynamics along tumor differentiation, we constructed pseudotime trajectories and evaluated their significance based on cluster ordering, as previously described.^68^ Gene and TF activity scores were smoothed along pseudotime using the loess function in R (span = 2). Heatmaps and trajectory plots were generated using the ggplot2 package in R to visualize the dynamic changes in gene and TF activities.

#### Genomic track profile

Chromatin accessibility of specific genomic regions was visualized by function CoveragePlot in Signac.

#### Pathway enrichment analysis

Pathway enrichment analysis was performed by first setting the default assay to “RNA” using the DefaultAssay function. Differentially expressed genes (DEGs) for each cluster were identified using the FindAllMarkers function with a minimum expression threshold of 10% (min.pct = 0.1) and a log fold change cutoff of 0.25. Gene set enrichment was conducted using the enricher function from the clusterProfiler^69^ package against the 50 hallmark gene sets curated in the Molecular Signatures Database (MSigDB).^70^

#### Spatial ATAC data visualization

Spatial pixel positions were initially determined from brightfield images using MATLAB 2020b (https://github.com/edicliuyang/Hip-lex_proteome). To visualize spatial distributions of chromatin features (e.g., unique nuclear fragments), fragment files were processed with ArchR^66^ (v1.0.1) to generate an ArchRProject, which was then imported into Seurat (v5.0.1) for tissue-level mapping. The pt.size.factor parameter was used to scale pixel size for optimal visualization. For gene activity scores or TF deviation scores, metadata from Signac objects were extracted and spatially projected onto tissue sections accordingly.

### Quantification and statistical analysis

Statistical analyses were performed using R (v4.3.2). Comparisons between two independent groups were assessed using unpaired two-tailed Mann–Whitney U tests. Differential chromatin accessibility and motif enrichment analyses were performed using the statistical procedures implemented in Seurat and Signac, respectively. Unless otherwise specified, *p* < 0.05 was considered statistically significant. Where statistical significance is indicated by asterisks, ∗∗∗∗ denotes *p* < 0.0001. Specific statistical tests and definitions of plotted summary measures are provided in the corresponding figure legends.

## Supplemental information

Document S1. Figures S1–S12 and Tables S1–S4

Data S1. Quality-control metrics and numerical source data underlying the indicated figures
