# Supplementary material for: Spatial epigenomics reveals stromal niche-associated regulatory programs across pancreatic neuroendocrine tumor grades
Source: iScience. 2026 Oct 19;29(11):117953. doi: 10.1016/j.isci.2026.117953 (PMC13695138; doi:10.1016/j.isci.2026.117953)
Supplement: Supplementary file 1 [file mmc1.pdf]

iScience, Volume *29*

Supplemental information

Spatial epigenomics reveals stromal niche-associated regulatory programs across pancreatic neuroendocrine tumor grades

Dejiang Wang, Xiangjun Di, Fu Gao, Gaocai Li, Liyun Lin, Shujie He, Di Zhang, Junyi Xu, Yu Jin, Yuanxin Liang, Michael Cecchini, Jill Lacy, John Kunstman, Pamela Kunz, Jing Du, and Yang Liu

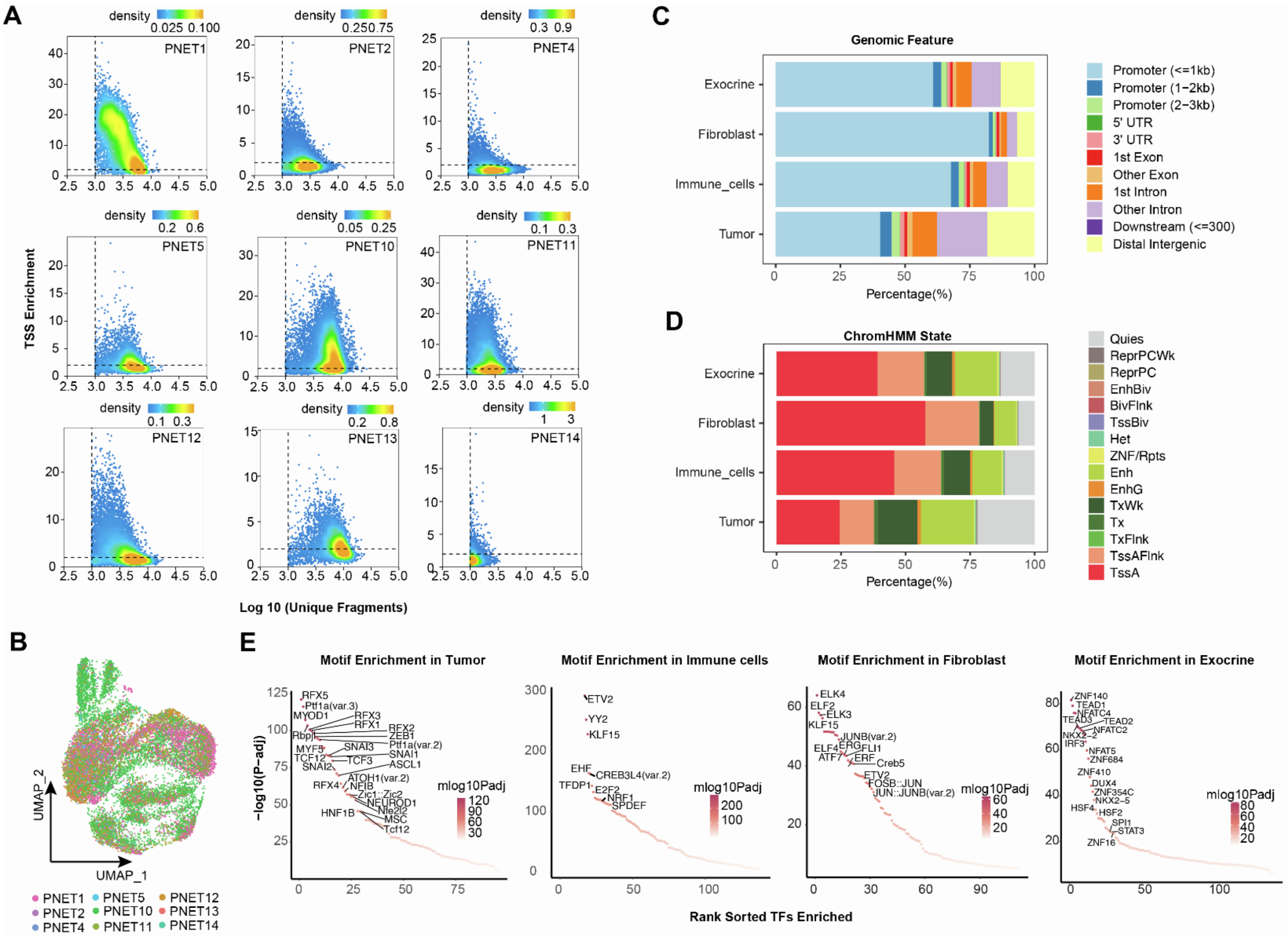

Fig. S1. Extended analysis of snATAC-seq profiling across nine NF-PanNET samples, Related to Figure 1. (**A**) Scatter plot of TSS enrichment score versus unique nuclear fragments per cell across nine NF-PanNETs, reflecting data quality and chromatin accessibility. (**B**) UMAP of snATAC-seq profiles across samples. (**C**) Genomic feature distribution of differentially accessible chromatin regions (DACRs) specific to each cell type. (**D**) ChromHMM-based chromatin state annotations for cell type-specific DACRs, categorized as follows: TssA (Active TSS), TssAFlnk (Flanking Active TSS), TxFlnk (Transcription at gene 5′ and 3′ ends), Tx (Strong transcription), TxWk (Weak transcription), EnhG (Genic enhancers), Enh (Enhancers), ZNF/Rpts (ZNF genes and repeats), Het (Heterochromatin), TssBiv (Bivalent/Poised TSS), BivFlnk (Flanking Bivalent TSS/Enhancer), EnhBiv (Bivalent Enhancer), ReprPC (Repressed PolyComb), ReprPCWk (Weak Repressed PolyComb), and Quies (Quiescent/Low signal). (**E**) Enriched transcription factor (TF) motifs identified in cell type-specific DACRs, suggesting distinct regulatory programs across cell populations.

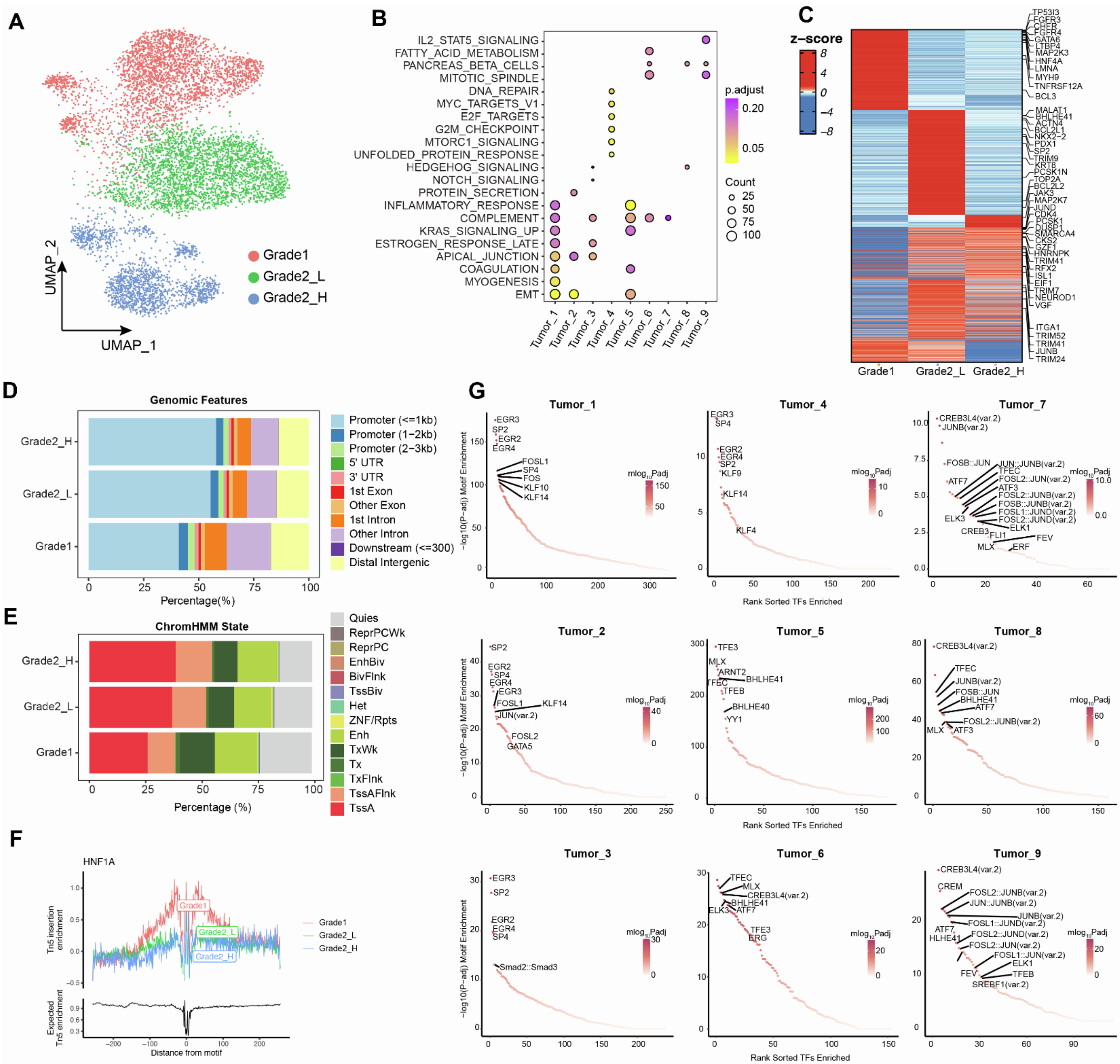

Fig. S2. Further analysis of chromatin accessibility and TF activities in tumor cells across grades, Related to Figure 2. (**A**) UMAP showing the annotated spatial-ATAC-seq profiles of tumor cells from different grades. (**B**) Enrichment of hallmark pathways derived from DACRs upregulated in each tumor subtype. Bubble size indicates gene count, and color represents the adjusted p-value. (**C**) Top differentially accessible peaks (DAPs) identified in tumor cells for each grade. Color indicates z-scaled average normalized accessibility. (**D**) Genomic feature distribution of tumor grade-specific DACRs. (**E**) ChromHMM annotation of tumor grade-specific DACRs. TssA, Active TSS; TssAFlnk, Flanking Active TSS; TxFlnk, Transcription at gene 5′ and 3′; Tx, Strong transcription; TxWk, Weak transcription; EnhG, Genic enhancers; Enh, Enhancers; ZNF/Rpts, ZNF genes and repeats; Het, Heterochromatin; TssBiv, Bivalent/Poised TSS; BivFlnk, Flanking Bivalent TSS/Enhancer; EnhBiv, Bivalent Enhancer; ReprPC, Repressed PolyComb; ReprPCWk, Weak Repressed PolyComb; Quies, Quiescent/Low. (**F**) HNF1A footprinting profiles across tumor grades. (**G**) Motif enrichment analysis of subtype-specific DACRs.

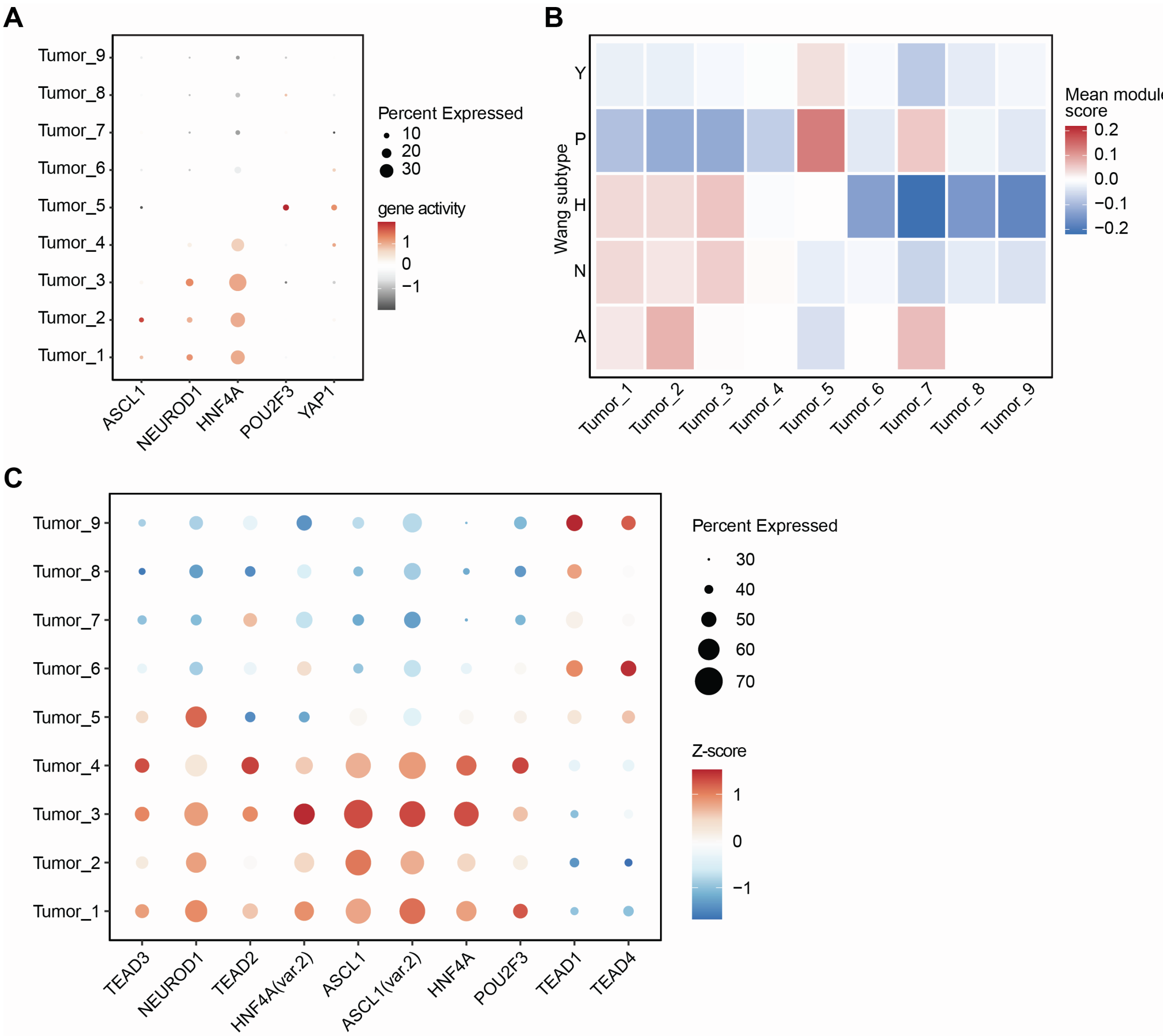

Fig. S3 Comparison of NF-PanNET tumor clusters with the five transcription factor–defined neuroendocrine carcinoma subtype programs reported by Wang et al, Related to Figure 2. (A) Dot plot showing gene activity of the five core subtype-associated transcription factors, ASCL1, NEUROD1, HNF4A, POU2F3, and YAP1, across the nine tumor clusters. Dot size represents the percentage of nuclei with detectable gene activity, and color indicates the scaled average gene activity within each cluster. (B) Heatmap showing the mean module scores of the Wang subtype-associated gene signatures across the nine tumor clusters. Rows correspond to the A-, N-, H-, P-, and Y-associated programs, and columns correspond to Tumor_1–Tumor_9. Red indicates relatively higher module scores and blue indicates relatively lower scores. (C) Dot plot showing chromVAR motif activity for selected transcription factors and TF families associated with the five subtype programs across the nine tumor clusters. Dot size represents the percentage of nuclei with detectable motif activity, and color indicates the scaled mean chromVAR deviation score.

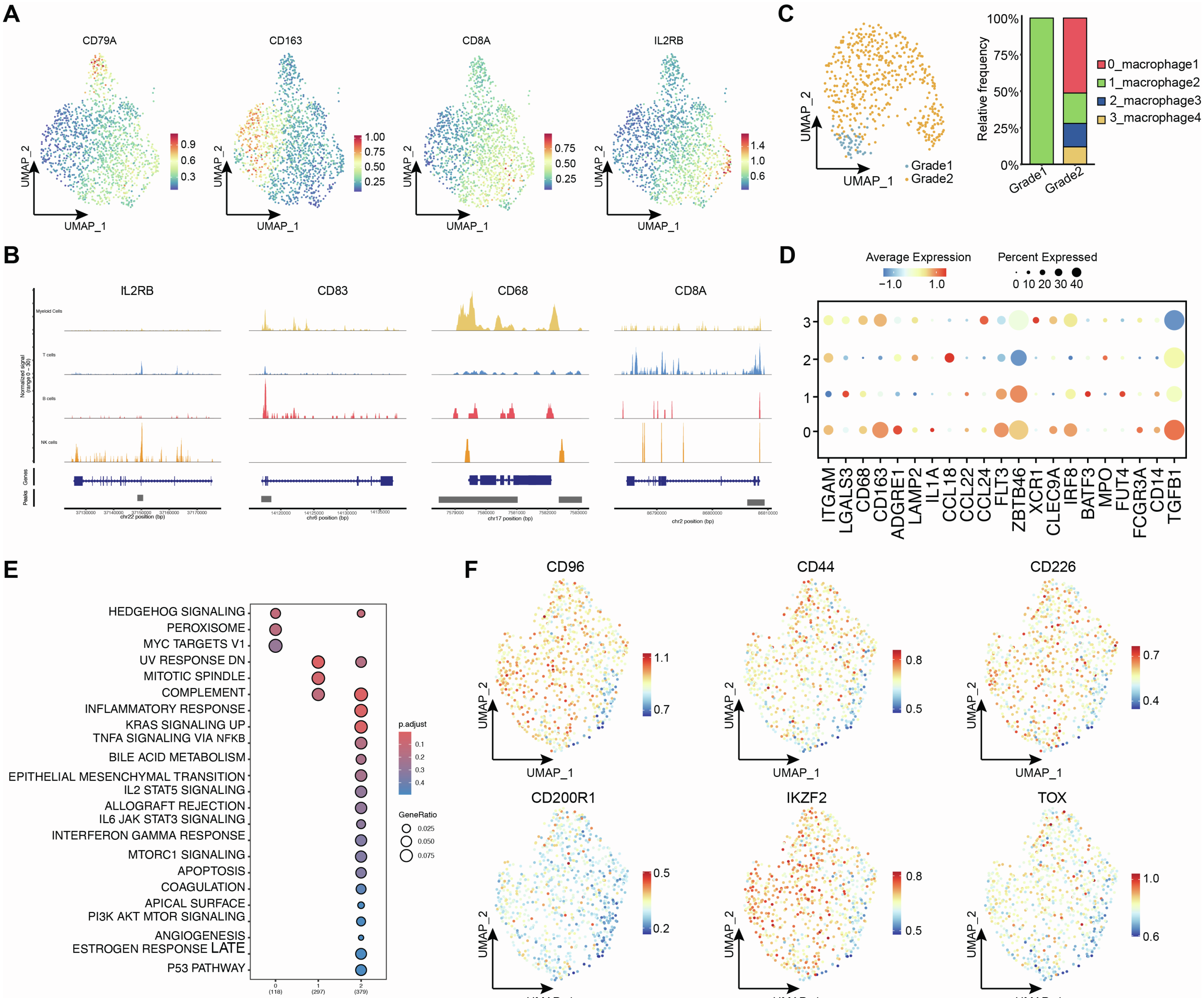

Fig. S4. Further analysis of chromatin accessibility and TF activities in immune cells, Related to Figure 4 and Figure 5. (**A**) UMAP of gene activity scores for selected marker genes across immune cell subtypes. (**B**) Coverage plots showing chromatin accessibility at marker gene loci for each immune cell subtype. (**C**) UMAP projection of macrophage cells colored by tumor grade. Each point represents one nucleus, with Grade 1 and Grade 2 cells shown separately (Left) and stacked bar plot showing the relative proportions of the four macrophage subclusters in Grade 1 and Grade 2 tumors (Right). (D) Dot plot of marker gene activity scores in macrophage subtypes. Dot size indicates the percentage of cells expressing the gene; color intensity reflects average gene activity scores (dark red: high, dark blue: low). (**E**) Enriched hallmark pathways derived from DACRs upregulated in each T cell subtype. Bubble size and color represent gene ratio and adjusted *p*-value, respectively. (**F**) UMAP projection of gene activity scores for selected marker genes across T cell subtypes.

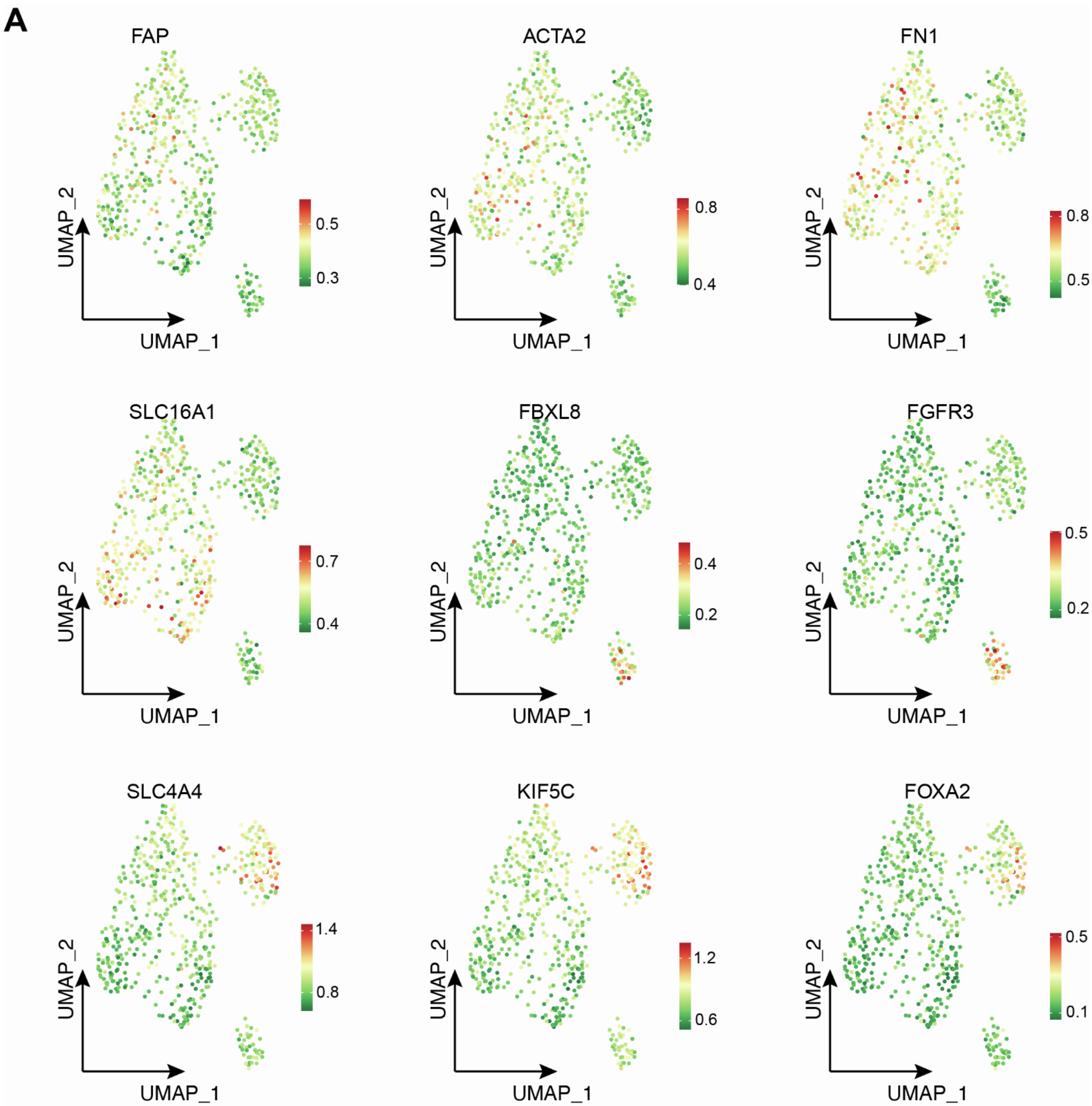

Fig. S5. Further analysis of chromatin accessibility and TF activities of CAFs, Related to Figure 6. UMAP projection of gene activity scores for selected marker genes across different CAF subtypes.

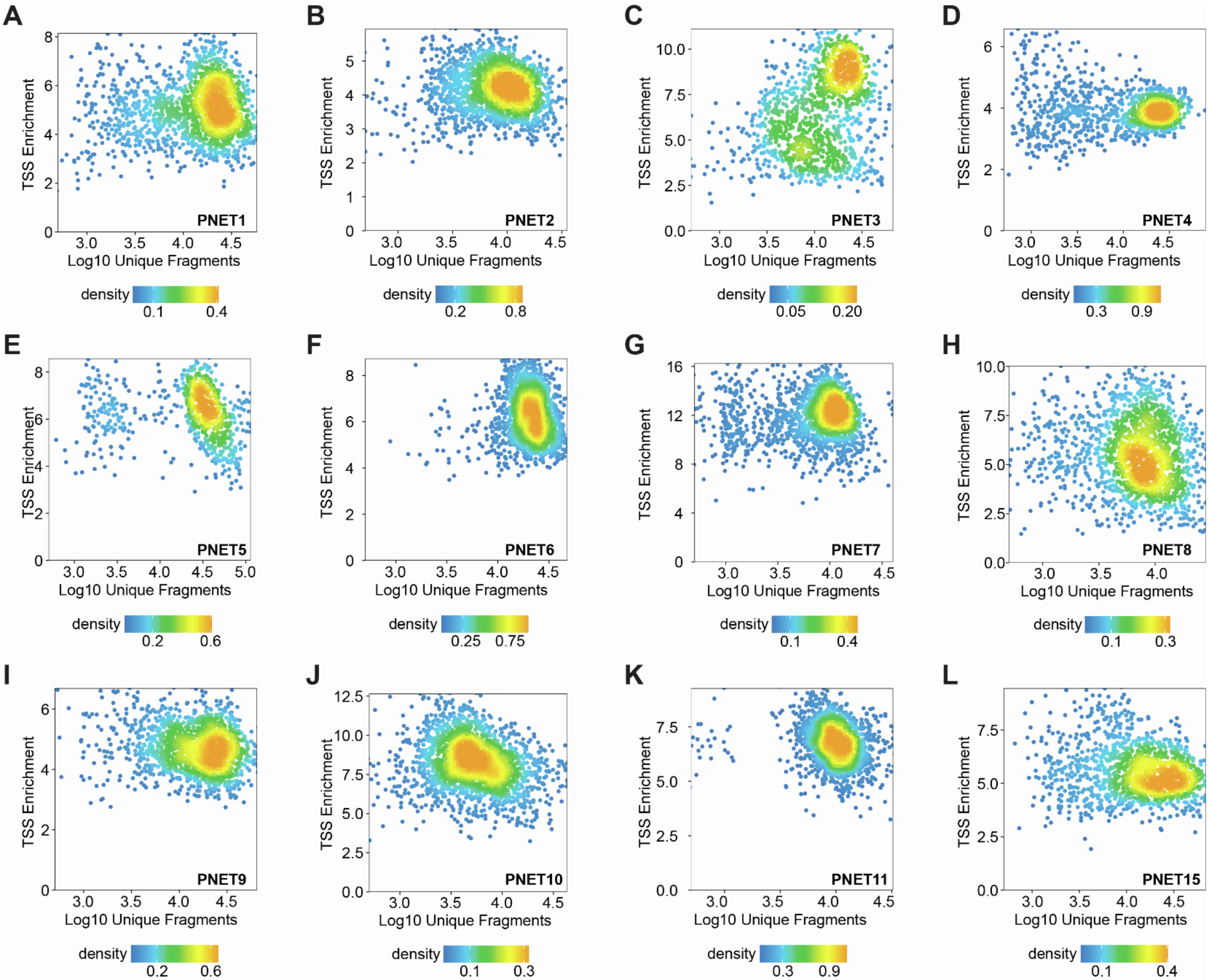

Fig. S6. TSS enrichment score versus unique nuclear fragments per cell for each spatial-ATAC-seq sample, Related to Figure 7. (**A**) PNET1; (**B**) PNET2; (**C**) PNET3; (**D**) PNET4; (**E**) PNET5; (**F**) PNET6; (**G**) PNET7; (**H**) PNET8; (**I**) PNET9; (**J**) PNET10; (**K**) PNET11; (**L**) PNET15.

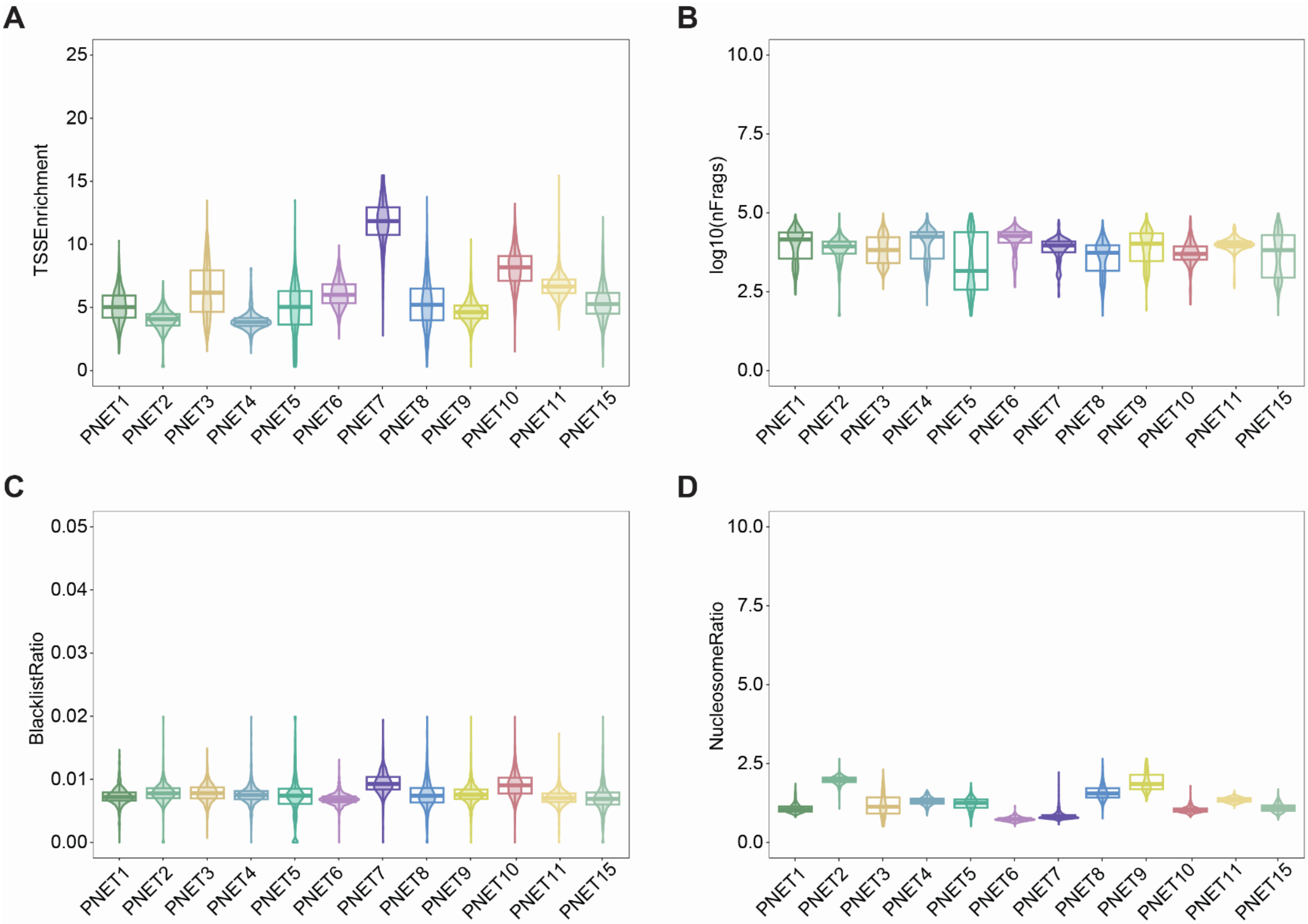

Fig. S7. Data quality metrics of spatial-ATAC-seq across different samples, Related to Figure 7. (**A**) TSS enrichment scores; (**B**) Unique nuclear fragment counts; (**C**) Blacklist ratios; (**D**) Nucleosome signal ratios. Violin plots show the distributions across spatial spots; boxes indicate the median and interquartile range, and whiskers extend to 1.5 × the interquartile range.

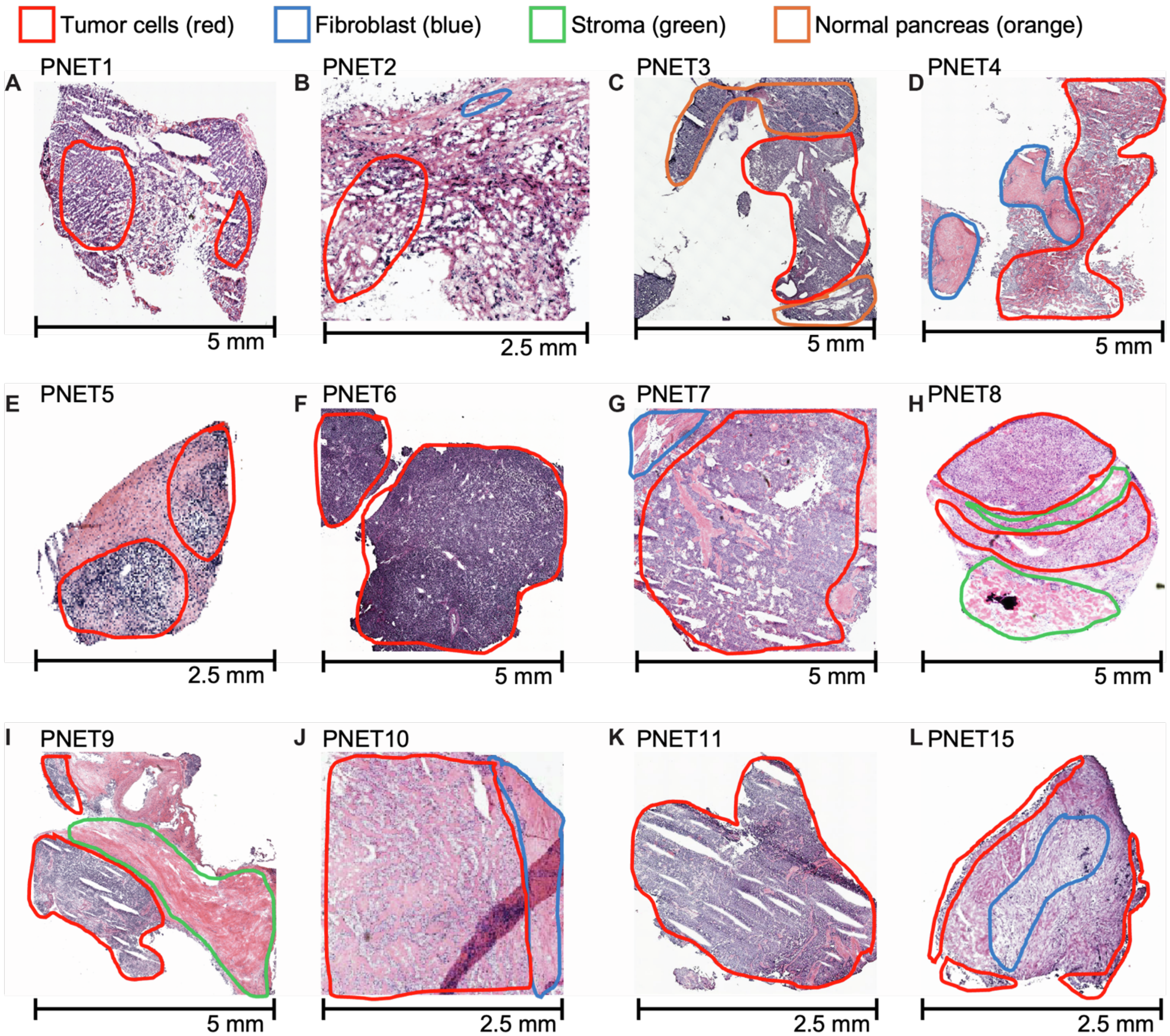

Fig. S8. Evaluating the tissue context of NF-PanNETs using spatial-ATAC-seq, Related to Figure 7. (**A-I**), H&E-stained images of tumor sections processed for spatial-ATAC-seq. Pathologist-annotated regions are highlighted: tumor (red), fibroblasts (blue), stroma (green), and normal pancreas (orange).

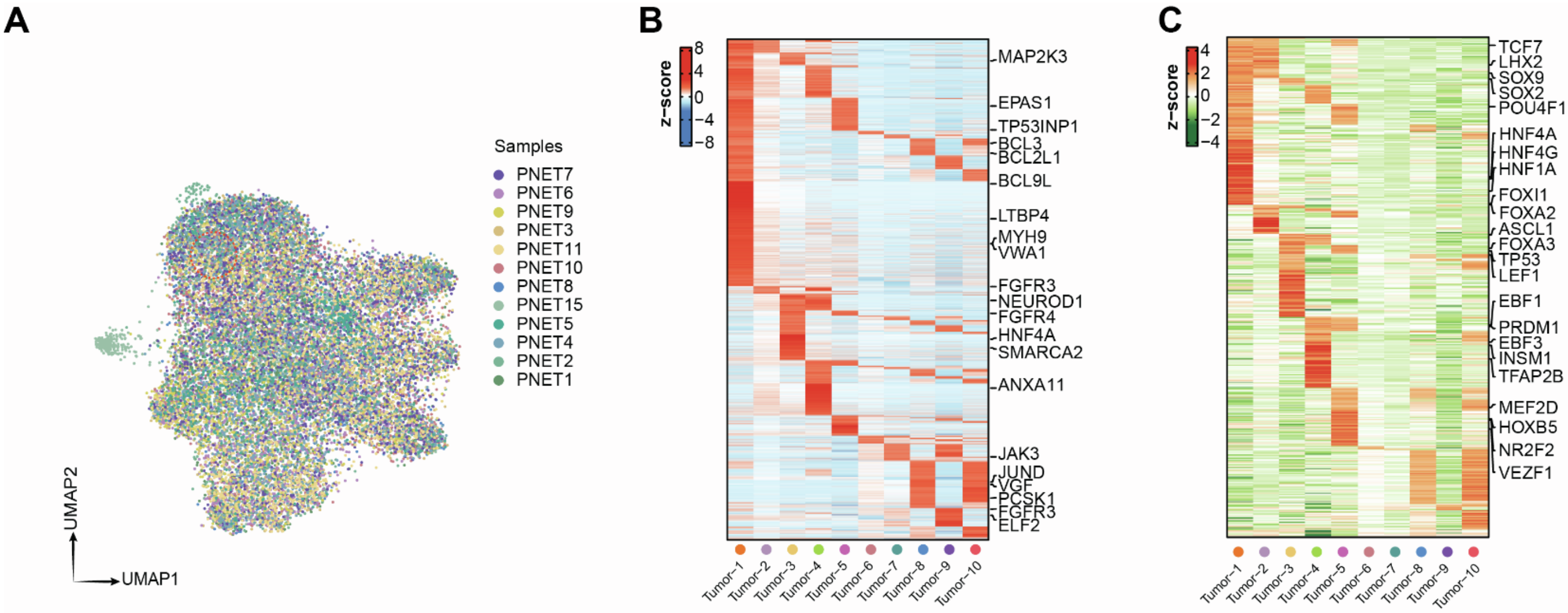

Fig. S9. Spatial chromatin accessibility and regulatory landscape of tumor subtypes in NF-PanNET, Related to Figure 7. (**A**) UMAP projection showing the annotated spatial-ATAC-seq profiles across different samples. (**B**) Top DACRs identified for each tumor subtype. Color represents the z-scaled average normalized chromatin accessibility signal within each subtype. (**C**) Differentially activated TFs across tumor subtypes. Color indicates the z-scaled average TF activity score for each subtype.

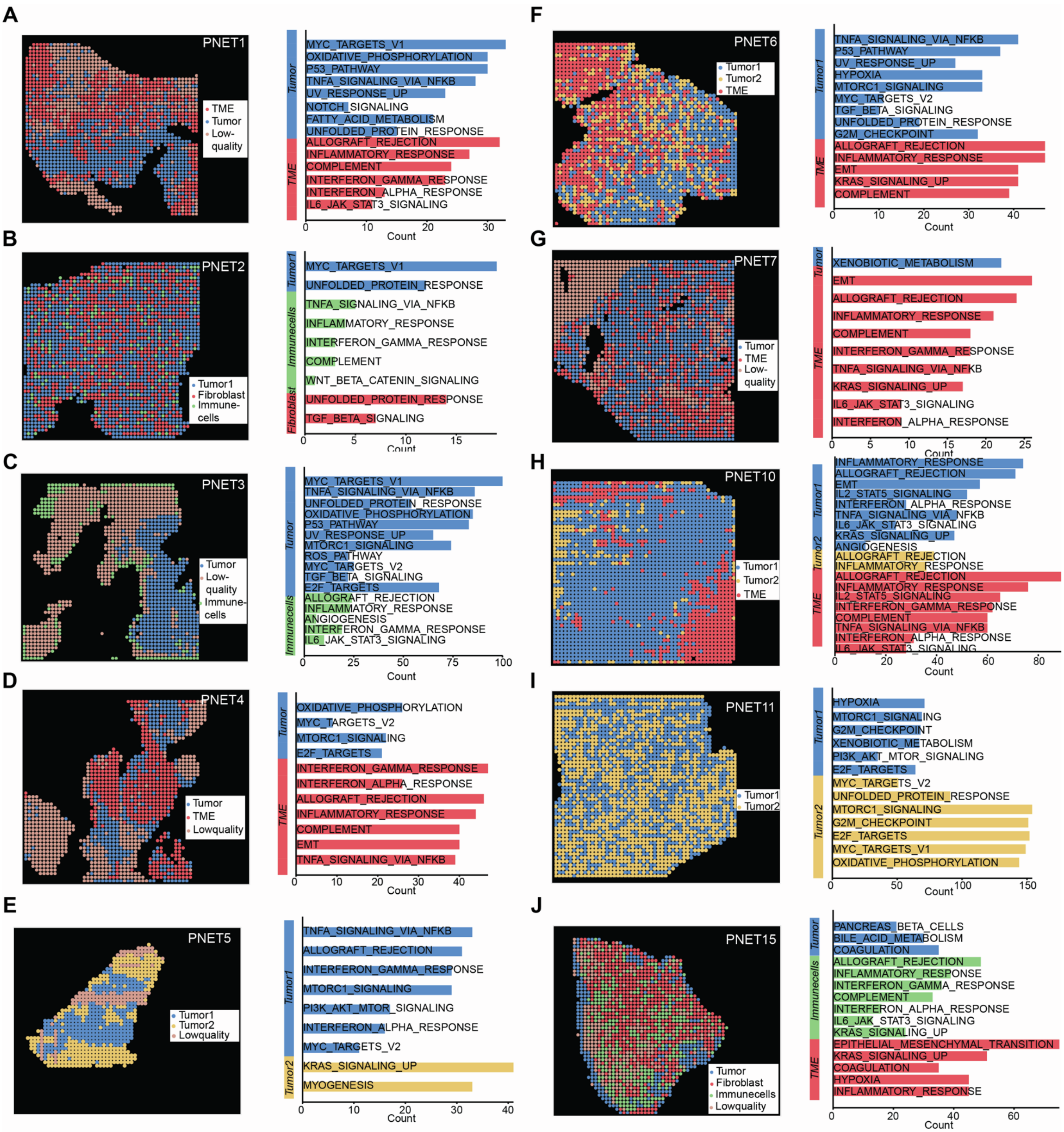

Fig. S10. Spatial mapping of chromatin accessibility-based clusters across NF-PanNET samples, Related to Figure 8. Unbiased clustering was performed based on chromatin accessibility profiles in each sample: PNET1 (**A**), PNET2 (**B**), PNET3 (**C**), PNET4 (**D**), PNET5 (**E**), PNET6 (**F**), PNET7 (**G**), PNET10 (**H**), PNET11 (**I**), and PNET15 (**J**).

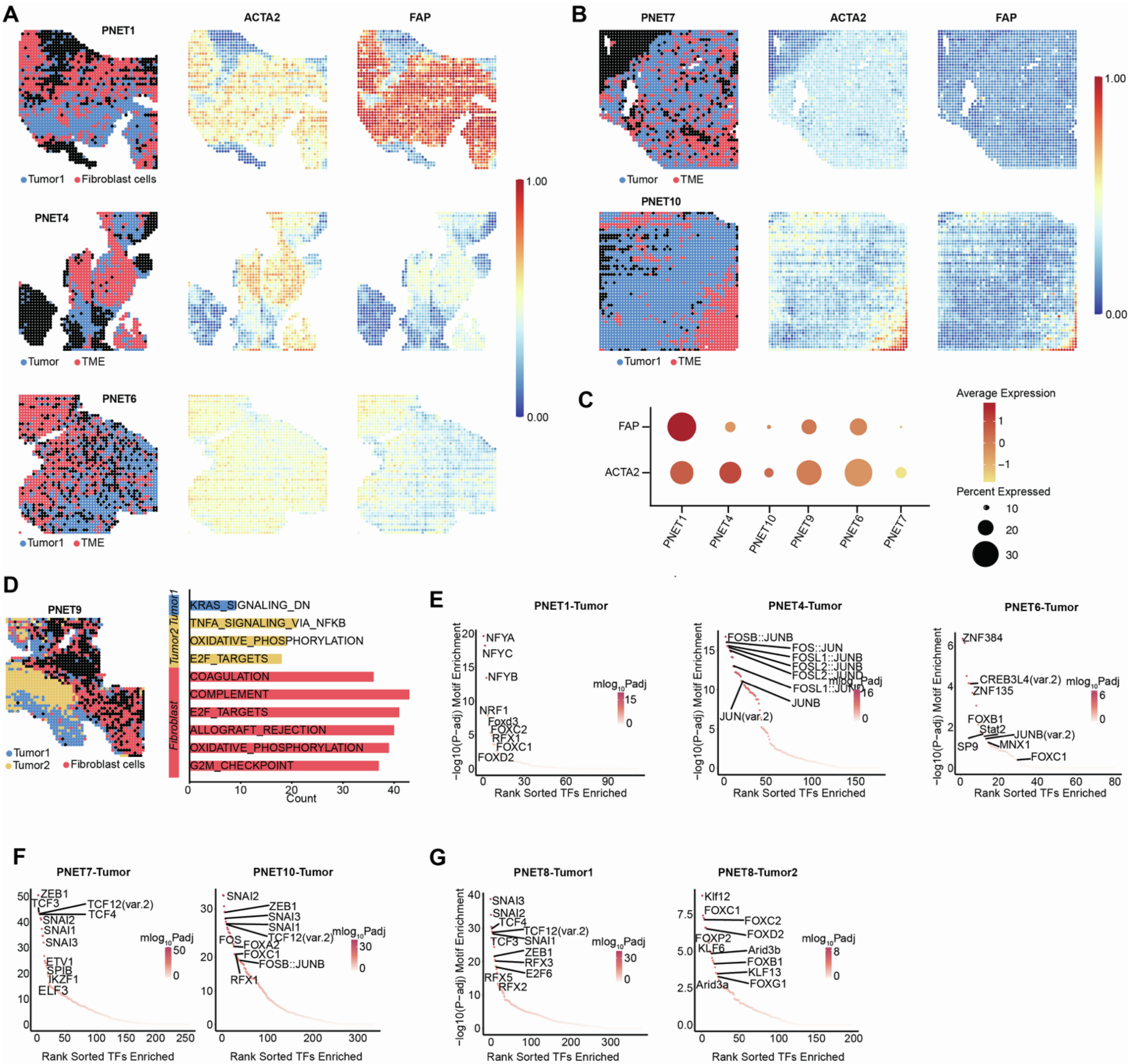

Fig. S11. Further analysis of spatial distribution of chromatin accessibility landscape in stroma and tumor, Related to Figure 8. (**A-B**) Spatial mapping of tumor and fibroblasts clusters (left), gene activity score of ACTA2 (middle), and gene activity score of FAP (right). (**C**) Dot plot showing the gene activity scores of CAF marker genes across samples. Dot size represents the percentage of cells with detected expression; color intensity indicates the average gene activity score per sample (dark red: higher activity; light red: lower activity). (**D**) Spatial mapping of tumor and fibroblasts clusters (left) and corresponding pathway enrichment analysis (right) in PNET9. (**E**) Enriched TF motifs in tumor-specific DACRs from samples PNET1 (left), PNET4 (middle), and PNET6 (right). (**F**) Enriched TF motifs in tumor-specific DACRs from samples PNET7 (left) and PNET10 (right). (**G**) Enriched TF motifs in tumor-specific DACRs from sample PNET8.

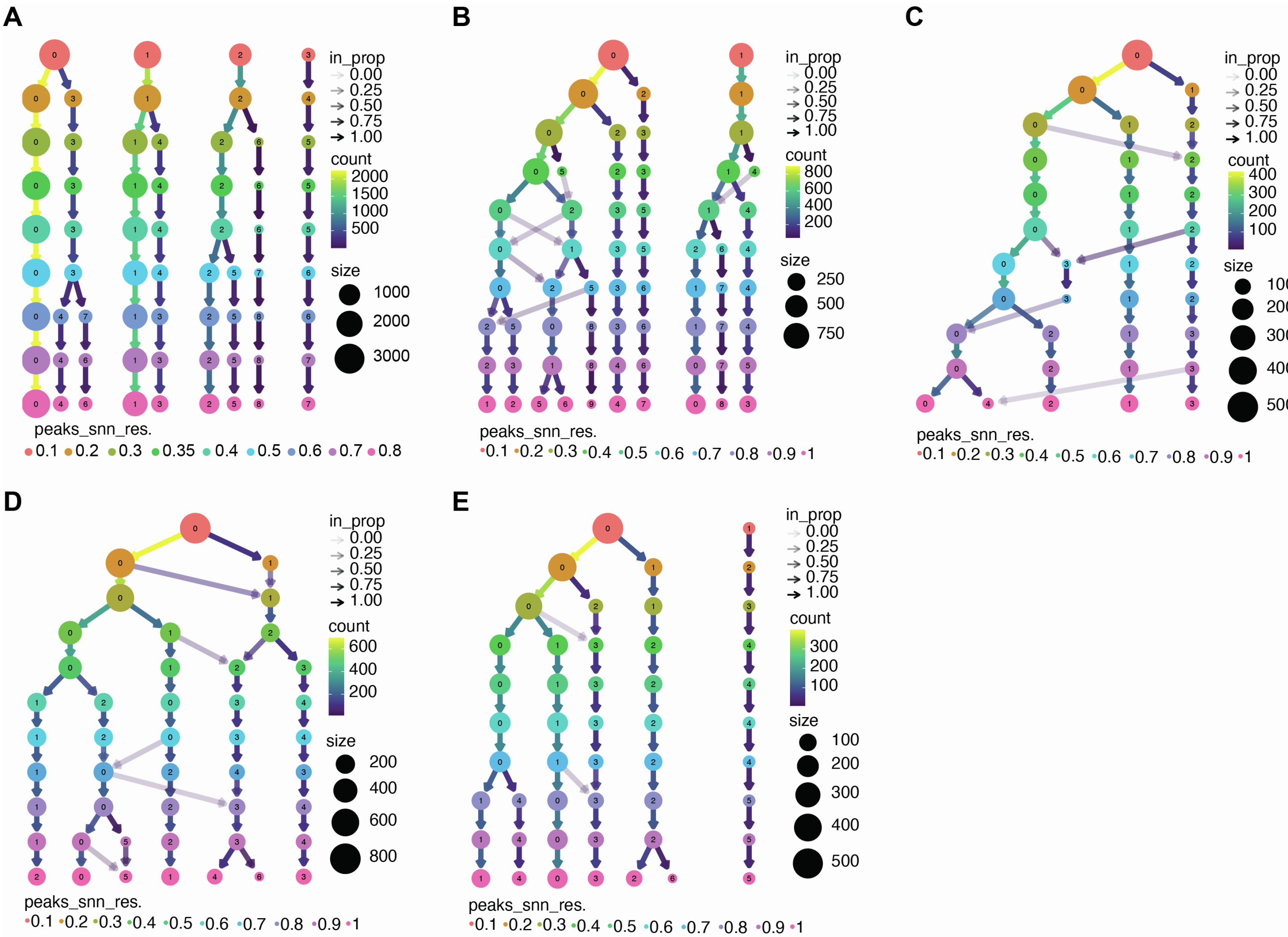

Fig. S12. Robustness assessment of graph-based clustering across resolution parameters, Related to Figure 2, Figure 4, Figure 5, and Figure 6. Clustering relationships across a range of resolution values were visualized using clustree for **(A) tumor cells, (B) immune cells, (C) myeloid cells, (D) T cells, and (E) cancer-associated fibroblasts**. Nodes represent clusters identified at each resolution, node size indicates the number of cells, and connecting edges indicate the proportion of cells retained between clusters at adjacent resolutions. Final resolutions were selected based on the persistence of cluster structure across neighboring resolutions, UMAP separation, consistency across samples, accessibility of canonical lineage markers, and avoidance of over-fragmentation. The selected resolutions were 0.6 for tumor cells, 0.3 for immune cells, 0.8 for myeloid cells, 0.4 for T cells, and 0.4 for cancer-associated fibroblasts.

Table S1. DNA oligos used for PCR and preparation of sequencing library, Related to Figure 1.

**Sequence Name**	**Sequence (5'-3')**
Ligation linker 1	AGTCGTACGCCGATGCGAAACATCGGCCAC
Ligation linker 2	CGAATGCTCTGGCCTCTCAAGCACGTGGAT
Tn5MErev	/5Phos/CTGTCTCTTATACACATCT
Tn5ME-A	TCGTCGGCAGCGTCAGATGTGTATAAGAGACAG
Tn5ME-B	/5Phos/CATCGGCGTACGACTAGATGTGTATAAGAGACAG
N501	AATGATACGGCGACCACCGAGATCTACACTAGATCGCTCGTCGGCAGCGTCA GATGTGTATAAGAGACAG
N502	AATGATACGGCGACCACCGAGATCTACACCTCTCTATTCGTCGGCAGCGTCA GATGTGTATAAGAGACAG
N701	CAAGCAGAAGACGGCATACGAGATTCGCCTTAGTCTCGTGGGCTCGGAGATG TGTATAAGAGACAGCAAGCGTTGGCTTCTCGCATCT
N702	CAAGCAGAAGACGGCATACGAGATCTAGTACGGTCTCGTGGGCTCGGAGAT GTGTATAAGAGACAGCAAGCGTTGGCTTCTCGCATCT
N703	CAAGCAGAAGACGGCATACGAGATTTCTGCCTGTCTCGTGGGCTCGGAGATG TGTATAAGAGACAGCAAGCGTTGGCTTCTCGCATCT
N704	CAAGCAGAAGACGGCATACGAGATGCTCAGGAGTCTCGTGGGCTCGGAGAT GTGTATAAGAGACAGCAAGCGTTGGCTTCTCGCATCT
N705	CAAGCAGAAGACGGCATACGAGATAGGAGTCCGTCTCGTGGGCTCGGAGAT GTGTATAAGAGACAGCAAGCGTTGGCTTCTCGCATCT
N706	CAAGCAGAAGACGGCATACGAGATCATGCCTAGTCTCGTGGGCTCGGAGATG TGTATAAGAGACAGCAAGCGTTGGCTTCTCGCATCT

Table S2. DNA barcode A sequences, Related to Figure 1.

Sequence Name	Sequence (5'-3')
Barcode A-1	/5Phos/AGGCCAGAGCATTCGAACGTGATGTGGCCGATGTTTCG
Barcode A-2	/5Phos/AGGCCAGAGCATTCGAAACATCGGTGGCCGATGTTTCG
Barcode A-3	/5Phos/AGGCCAGAGCATTCGATGCCTAAGTGGCCGATGTTTCG
Barcode A-4	/5Phos/AGGCCAGAGCATTCGAGTGGTCAGTGGCCGATGTTTCG
Barcode A-5	/5Phos/AGGCCAGAGCATTCGACCACTGTGTGGCCGATGTTTCG
Barcode A-6	/5Phos/AGGCCAGAGCATTCGACATTGGCGTGGCCGATGTTTCG
Barcode A-7	/5Phos/AGGCCAGAGCATTCGCAGATCTGGTGGCCGATGTTTCG
Barcode A-8	/5Phos/AGGCCAGAGCATTCGCATCAAGTGTGGCCGATGTTTCG
Barcode A-9	/5Phos/AGGCCAGAGCATTCGCGCTGATCGTGGCCGATGTTTCG
Barcode A-10	/5Phos/AGGCCAGAGCATTCGACAAGCTAGTGGCCGATGTTTCG
Barcode A-11	/5Phos/AGGCCAGAGCATTCGCTGTAGCCGTGGCCGATGTTTCG
Barcode A-12	/5Phos/AGGCCAGAGCATTCGAGTACAAGGTGGCCGATGTTTCG
Barcode A-13	/5Phos/AGGCCAGAGCATTCGAACAACCAGTGGCCGATGTTTCG
Barcode A-14	/5Phos/AGGCCAGAGCATTCGAACCGAGAGTGGCCGATGTTTCG
Barcode A-15	/5Phos/AGGCCAGAGCATTCGAACGCTTAGTGGCCGATGTTTCG
Barcode A-16	/5Phos/AGGCCAGAGCATTCGAAGACGGAGTGGCCGATGTTTCG
Barcode A-17	/5Phos/AGGCCAGAGCATTCGAAGGTACAGTGGCCGATGTTTCG
Barcode A-18	/5Phos/AGGCCAGAGCATTCGACACAGAAGTGGCCGATGTTTCG
Barcode A-19	/5Phos/AGGCCAGAGCATTCGACAGCAGAGTGGCCGATGTTTCG
Barcode A-20	/5Phos/AGGCCAGAGCATTCGACCTCCAAGTGGCCGATGTTTCG
Barcode A-21	/5Phos/AGGCCAGAGCATTCGACGCTCGAGTGGCCGATGTTTCG
Barcode A-22	/5Phos/AGGCCAGAGCATTCGACGTATCAGTGGCCGATGTTTCG
Barcode A-23	/5Phos/AGGCCAGAGCATTCGACTATGCAGTGGCCGATGTTTCG
Barcode A-24	/5Phos/AGGCCAGAGCATTCGAGAGTCAAGTGGCCGATGTTTCG
Barcode A-25	/5Phos/AGGCCAGAGCATTCGAGATCGCAGTGGCCGATGTTTCG
Barcode A-26	/5Phos/AGGCCAGAGCATTCGAGCAGGAAGTGGCCGATGTTTCG
Barcode A-27	/5Phos/AGGCCAGAGCATTCGAGTCACTAGTGGCCGATGTTTCG
Barcode A-28	/5Phos/AGGCCAGAGCATTCGATCCTGTAGTGGCCGATGTTTCG
Barcode A-29	/5Phos/AGGCCAGAGCATTCGATTGAGGAGTGGCCGATGTTTCG
Barcode A-30	/5Phos/AGGCCAGAGCATTCGCAACCACAGTGGCCGATGTTTCG
Barcode A-31	/5Phos/AGGCCAGAGCATTCGGACTAGTAGTGGCCGATGTTTCG
Barcode A-32	/5Phos/AGGCCAGAGCATTCGCAATGGAAGTGGCCGATGTTTCG
Barcode A-33	/5Phos/AGGCCAGAGCATTCGCACTTCGAGTGGCCGATGTTTCG
Barcode A-34	/5Phos/AGGCCAGAGCATTCGCAGCGTTAGTGGCCGATGTTTCG
Barcode A-35	/5Phos/AGGCCAGAGCATTCGCATACCAAGTGGCCGATGTTTCG
Barcode A-36	/5Phos/AGGCCAGAGCATTCGCCAGTTCAGTGGCCGATGTTTCG
Barcode A-37	/5Phos/AGGCCAGAGCATTCGCCGAAGTAGTGGCCGATGTTTCG
Barcode A-38	/5Phos/AGGCCAGAGCATTCGCCGTGAGAGTGGCCGATGTTTCG

Barcode A-39	/5Phos/AGGCCAGAGCATTCGCCTCCTGAGTGGCCGATGTTTCG
Barcode A-40	/5Phos/AGGCCAGAGCATTCGCGAACTTAGTGGCCGATGTTTCG
Barcode A-41	/5Phos/AGGCCAGAGCATTCGCGACTGGAGTGGCCGATGTTTCG
Barcode A-42	/5Phos/AGGCCAGAGCATTCGCGCATACAGTGGCCGATGTTTCG
Barcode A-43	/5Phos/AGGCCAGAGCATTCGCTCAATGAGTGGCCGATGTTTCG
Barcode A-44	/5Phos/AGGCCAGAGCATTCGCTGAGCCAGTGGCCGATGTTTCG
Barcode A-45	/5Phos/AGGCCAGAGCATTCGCTGGCATAGTGGCCGATGTTTCG
Barcode A-46	/5Phos/AGGCCAGAGCATTCGGAATCTGAGTGGCCGATGTTTCG
Barcode A-47	/5Phos/AGGCCAGAGCATTCGCAAGACTAGTGGCCGATGTTTCG
Barcode A-48	/5Phos/AGGCCAGAGCATTCGGAGCTGAAGTGGCCGATGTTTCG
Barcode A-49	/5Phos/AGGCCAGAGCATTCGGATAGACAGTGGCCGATGTTTCG
Barcode A-50	/5Phos/AGGCCAGAGCATTCGGCCACATAGTGGCCGATGTTTCG

Table S3. DNA barcode B sequences, Related to Figure 1.

Sequence Name	Sequence (5'-3')
barcode B-1	CAAGCGTTGGCTTCTCGCATCTNNNNNNNNNNAACGTGATATCCACGTGCTTGAG
barcode B-2	CAAGCGTTGGCTTCTCGCATCTNNNNNNNNNNAAACATCGATCCACGTGCTTGAG
barcode B-3	CAAGCGTTGGCTTCTCGCATCTNNNNNNNNNNATGCCTAAATCCACGTGCTTGAG
barcode B-4	CAAGCGTTGGCTTCTCGCATCTNNNNNNNNNNAGTGGTCAATCCACGTGCTTGAG
barcode B-5	CAAGCGTTGGCTTCTCGCATCTNNNNNNNNNNACCACTGTATCCACGTGCTTGAG
barcode B-6	CAAGCGTTGGCTTCTCGCATCTNNNNNNNNNNACATTGGCATCCACGTGCTTGAG
barcode B-7	CAAGCGTTGGCTTCTCGCATCTNNNNNNNNNNCAGATCTGATCCACGTGCTTGAG
barcode B-8	CAAGCGTTGGCTTCTCGCATCTNNNNNNNNNNCATCAAGTATCCACGTGCTTGAG
barcode B-9	CAAGCGTTGGCTTCTCGCATCTNNNNNNNNNNCGCTGATCATCCACGTGCTTGAG
barcode B-10	CAAGCGTTGGCTTCTCGCATCTNNNNNNNNNNACAAGCTAATCCACGTGCTTGAG
barcode B-11	CAAGCGTTGGCTTCTCGCATCTNNNNNNNNNNCTGTAGCCATCCACGTGCTTGAG
barcode B-12	CAAGCGTTGGCTTCTCGCATCTNNNNNNNNNNAGTACAAGATCCACGTGCTTGAG
barcode B-13	CAAGCGTTGGCTTCTCGCATCTNNNNNNNNNNAACAACCAATCCACGTGCTTGAG
barcode B-14	CAAGCGTTGGCTTCTCGCATCTNNNNNNNNNNAACCGAGAATCCACGTGCTTGAG
barcode B-15	CAAGCGTTGGCTTCTCGCATCTNNNNNNNNNNAACGCTTAATCCACGTGCTTGAG
barcode B-16	CAAGCGTTGGCTTCTCGCATCTNNNNNNNNNNAAGACGGAATCCACGTGCTTGAG
barcode B-17	CAAGCGTTGGCTTCTCGCATCTNNNNNNNNNNAAGGTACAATCCACGTGCTTGAG
barcode B-18	CAAGCGTTGGCTTCTCGCATCTNNNNNNNNNNACACAGAAATCCACGTGCTTGAG
barcode B-19	CAAGCGTTGGCTTCTCGCATCTNNNNNNNNNNACAGCAGAATCCACGTGCTTGAG
barcode B-20	CAAGCGTTGGCTTCTCGCATCTNNNNNNNNNNACCTCCAAATCCACGTGCTTGAG
barcode B-21	CAAGCGTTGGCTTCTCGCATCTNNNNNNNNNNACGCTCGAATCCACGTGCTTGAG
barcode B-22	CAAGCGTTGGCTTCTCGCATCTNNNNNNNNNNACGTATCAATCCACGTGCTTGAG
barcode B-23	CAAGCGTTGGCTTCTCGCATCTNNNNNNNNNNACTATGCAATCCACGTGCTTGAG
barcode B-24	CAAGCGTTGGCTTCTCGCATCTNNNNNNNNNNAGAGTCAAATCCACGTGCTTGAG
barcode B-25	CAAGCGTTGGCTTCTCGCATCTNNNNNNNNNNAGATCGCAATCCACGTGCTTGAG
barcode B-26	CAAGCGTTGGCTTCTCGCATCTNNNNNNNNNNAGCAGGAAATCCACGTGCTTGAG
barcode B-27	CAAGCGTTGGCTTCTCGCATCTNNNNNNNNNNAGTCACTAATCCACGTGCTTGAG
barcode B-28	CAAGCGTTGGCTTCTCGCATCTNNNNNNNNNNATCCTGTAATCCACGTGCTTGAG
barcode B-29	CAAGCGTTGGCTTCTCGCATCTNNNNNNNNNNATTGAGGAATCCACGTGCTTGAG
barcode B-30	CAAGCGTTGGCTTCTCGCATCTNNNNNNNNNNCAACCACAATCCACGTGCTTGAG
barcode B-31	CAAGCGTTGGCTTCTCGCATCTNNNNNNNNNNGACTAGTAATCCACGTGCTTGAG
barcode B-32	CAAGCGTTGGCTTCTCGCATCTNNNNNNNNNNCAATGGAAATCCACGTGCTTGAG
barcode B-33	CAAGCGTTGGCTTCTCGCATCTNNNNNNNNNNCACTTCGAATCCACGTGCTTGAG
barcode B-34	CAAGCGTTGGCTTCTCGCATCTNNNNNNNNNNCAGCGTTAATCCACGTGCTTGAG
barcode B-35	CAAGCGTTGGCTTCTCGCATCTNNNNNNNNNNCATACCAAATCCACGTGCTTGAG
barcode B-36	CAAGCGTTGGCTTCTCGCATCTNNNNNNNNNNCCAGTTCAATCCACGTGCTTGAG
barcode B-37	CAAGCGTTGGCTTCTCGCATCTNNNNNNNNNNCCGAAGTAATCCACGTGCTTGAG
barcode B-38	CAAGCGTTGGCTTCTCGCATCTNNNNNNNNNNCCGTGAGAATCCACGTGCTTGAG

barcode B-39	CAAGCGTTGGCTTCTCGCATCTNNNNNNNNNNCCTCCTGAATCCACGTGCTTGAG
barcode B-40	CAAGCGTTGGCTTCTCGCATCTNNNNNNNNNNCGAACTTAATCCACGTGCTTGAG
barcode B-41	CAAGCGTTGGCTTCTCGCATCTNNNNNNNNNNCGACTGGAATCCACGTGCTTGAG
barcode B-42	CAAGCGTTGGCTTCTCGCATCTNNNNNNNNNNCGCATACAATCCACGTGCTTGAG
barcode B-43	CAAGCGTTGGCTTCTCGCATCTNNNNNNNNNNCTCAATGAATCCACGTGCTTGAG
barcode B-44	CAAGCGTTGGCTTCTCGCATCTNNNNNNNNNNCTGAGCCAATCCACGTGCTTGAG
barcode B-45	CAAGCGTTGGCTTCTCGCATCTNNNNNNNNNNCTGGCATAATCCACGTGCTTGAG
barcode B-46	CAAGCGTTGGCTTCTCGCATCTNNNNNNNNNNGAATCTGAATCCACGTGCTTGAG
barcode B-47	CAAGCGTTGGCTTCTCGCATCTNNNNNNNNNNCAAGACTAATCCACGTGCTTGAG
barcode B-48	CAAGCGTTGGCTTCTCGCATCTNNNNNNNNNNGAGCTGAAATCCACGTGCTTGAG
barcode B-49	CAAGCGTTGGCTTCTCGCATCTNNNNNNNNNNGATAGACAATCCACGTGCTTGAG
barcode B-50	CAAGCGTTGGCTTCTCGCATCTNNNNNNNNNNGCCACATAATCCACGTGCTTGAG

Table S4. Demographic and clinical characteristics of human PanNET samples, Related to Figure 1.

Sample	Gender	Age	Hispanic/Latina	Grade
PNET1	Male	54	N	1
PNET2	Female	59	N	1
PNET3	Male	57	N	1
PNET4	Male	70	N	1
PNET5	Male	60	N	1
PNET6	Male	61	Y	1
PNET7	Male	48	Y	2
PNET8	Male	50	N	2
PNET9	Male	70	N	2
PNET10	Female	61	N	2
PNET11	Female	78	N	2
PNET12	Female	55	N	2
PNET13	Female	61	N	2
PNET14	Female	54	N	2
PNET15	Female	55	N	3
